# Integrated Metabolomics and Morphogenesis Reveal Volatile Signaling of the Nematode-Trapping Fungus Arthrobotrys oligospora

**DOI:** 10.1128/AEM.02749-17

**Published:** 2018-04-16

**Authors:** Bai-Le Wang, Yong-Hong Chen, Jia-Ning He, Hua-Xi Xue, Ni Yan, Zhi-Jun Zeng, Joan W. Bennett, Ke-Qin Zhang, Xue-Mei Niu

**Affiliations:** a State Key Laboratory for Conservation and Utilization of Bio-Resources & Key Laboratory for Microbial Resources of the Ministry of Education, School of Life Sciences, Yunnan University, Kunming, People's Republic of China; b Department of Plant Biology, Rutgers University, New Brunswick, New Jersey, USA; University of Bayreuth

**Keywords:** nematode-trapping fungi, *Arthrobotrys oligospora*, metabolic adaptation, pathogenicity, volatile organic compounds, VOCs

## Abstract

The adjustment of metabolic patterns is fundamental to fungal biology and plays vital roles in adaptation to diverse ecological challenges. Nematode-trapping fungi can switch their lifestyle from saprophytic to pathogenic by developing specific trapping devices induced by nematodes to infect their prey as a response to nutrient depletion in nature. However, the chemical identity of the specific fungal metabolites used during the switch remains poorly understood. We hypothesized that these important signal molecules might be volatile in nature. Gas chromatography-mass spectrometry was used to carry out comparative analysis of fungal metabolomics during the saprophytic and pathogenic lifestyles of the model species Arthrobotrys oligospora. Two media commonly used in research on this species, cornmeal agar (CMA) and potato dextrose agar (PDA), were chosen for use in this study. The fungus produced a small group of volatile furanone and pyrone metabolites that were associated with the switch from the saprophytic to the pathogenic stage. A. oligospora fungi grown on CMA tended to produce more traps and employ attractive furanones to improve the utilization of traps, while fungi grown on PDA developed fewer traps and used nematode-toxic furanone metabolites to compensate for insufficient traps. Another volatile pyrone metabolite, maltol, was identified as a morphological regulator for enhancing trap formation. Deletion of the gene *AOL_s00079g496* in A. oligospora led to increased amounts of the furanone attractant (2-fold) in mutants and enhanced the attractive activity (1.5-fold) of the fungus, while it resulted in decreased trap formation. This investigation provides new insights regarding the comprehensive tactics of fungal adaptation to environmental stress, integrating both morphological and metabolomic mechanisms.

**IMPORTANCE** Nematode-trapping fungi are a unique group of soil-living fungi that can switch from the saprophytic to the pathogenic lifestyle once they come into contact with nematodes as a response to nutrient depletion. In this study, we investigated the metabolic response during the switch and the key types of metabolites involved in the interaction between fungi and nematodes. Our findings indicate that A. oligospora develops multiple and flexible metabolic tactics corresponding to different morphological responses to nematodes. A. oligospora can use similar volatile furanone and pyrone metabolites with different ecological functions to help capture nematodes in the fungal switch from the saprophytic to the pathogenic lifestyle. Furthermore, studies with A. oligospora mutants with increased furanone and pyrone metabolites confirmed the results. This investigation reveals the importance of volatile signaling in the comprehensive tactics used by nematode-trapping fungi, integrating both morphological and metabolomic mechanisms.

## INTRODUCTION

Nematode-trapping fungi (NTF) can detect the presence of nematodes and develop specialized mycelial trap devices to infect and consume prey as a response to nutrient depletion (1–4). These fungi are broadly distributed in terrestrial and aquatic ecosystems, and more than 200 species from the phyla Ascomycota, Basidiomycota, and Zygomycota have been described. Their role as natural enemies of parasitic nematodes makes them attractive as biocontrol agents; moreover, their unique ability to switch between saprophytic and parasitic lifestyles is of great interest in basic ecological research (5, 6).

Direct physical contact with living nematodes has been assumed to be the crucial biotic factor necessary to induce the trap formation of NTF (7, 8). Traps are regarded as the key morphological indication of the switch from the saprophytic to the pathogenic lifestyle for NTF (9–15). The nematodes not only induce the formation of fungal traps, but once they are trapped, they also serve as a food source (3). Considerable progress has been made in our understanding of the evolution and molecular mechanisms of fungal trap formation at the genomic, proteomic, and transcriptomic levels (14, 16). When nematodes induce the formation of trapping devices, multiple fungal signal transduction pathways are activated and the downstream genes associated with energy metabolism and biosynthesis of the cell wall and adhesive proteins involved in trap formation are regulated (7, 17).

Interestingly, NTF need an organic energy source other than nematodes in order to remain in an active nematophagous state (18, 19). Previous studies have found that cornmeal agar (CMA) and potato dextrose agar (PDA) are among the best media for keeping the nematophagous activities of NTF and the most extensively used by experimentalists to observe the trap formation induced by nematodes (8, 12, 14, 16–18). The composition of the growth medium is important because fungi produce different numbers of traps and have different nematocidal activities when grown on CMA or PDA. However, the chemical identity of the signaling molecules responsible for these differential responses has remained unclear.

It has long been assumed that traps are not the only weapons that NTF use to infect nematodes. In 1955, Duddington (19) and Shepherd (20) suggested that NTF could yield an unknown metabolite, nematotoxin, to paralyze or kill nematodes because they found that the infected nematodes became inactive before the infection bulb had completely developed. Later, in 1963, Olthof and Estey reported that the filtrates from NTF-parasitized nematodes contained the unstable nematode-inactivating substance (21). In 1994, linoleic acid was reported to be a putative nematicidal compound from several nematophagous fungi (22). At the same time, a unique class of hybrid oligosporon metabolites found to be chemotaxonomic markers was reported from different strains of a model species of Arthrobotrys oligospora isolated from The Netherlands (23), from Australia (24), and, later, from China (25, 26). These known metabolites from A. oligospora are nonvolatile compounds and exhibit several biological activities, including moderate antibacterial properties and significant autoregulatory effects on the formation of conidiophores and hyphal fusions in A. oligospora (26, 27). However, the chemical identity of the metabolites involved in the lifestyle switch from the saprophytic to the pathogenic phase has remained cryptic. We hypothesized that these signals might be volatile in nature and used gas chromatography (GC)-mass spectrometry (MS) in our analyses.

A. oligospora is commonly found in soils from diverse ecological habits and has emerged as the model species for nematode-trapping fungi (5, 27). Under limited conditions, A. oligospora can form three-dimensional (3D) traps in direct contact with nematodes. In order to obtain information on the morphological and metabolic changes in A. oligospora, two common media, CMA and PDA, were used in our analyses to investigate medium-specific metabolic features, as well as to illuminate general aspects of A. oligospora metabolism. There has been no previous report about the response of the fungus just before the fungus starts to form predatory traps via direct physical contact with nematodes, so a bioassay of nondirect contact between the fungus and living/dead nematodes was also performed. Dead nematodes were included in the non-direct-contact bioassay in order to further evaluate if the fungus could make different responses to the approaching living and dead nematodes. The time course designs over short-term intervals have provided successive snapshots of the morphological and metabolic status of A. oligospora (model strain YMF1.01883) in response to a shift from the absence of the nematodes to the presence of nematodes. GC-MS analysis was performed for metabolite profiling to determine the similarities and differences in temporal metabolite responses, and we have identified several volatile compounds that exhibit medium-specific responses during the induction of traps in response to the presence of nematodes.

## RESULTS

### Differences in hyphal morphogenesis of A. oligospora on CMA and PDA in response to nematodes.

**FIG 1:**
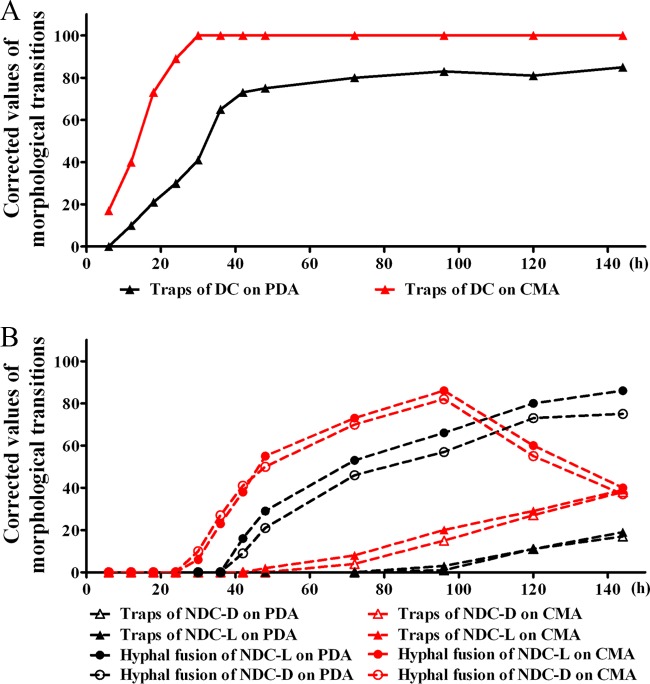
Morphological responses of A. oligospora grown on two different media, CMA and PDA, to living nematodes (L) or dead nematodes (D) under two different modes of contact, direct contact (DC) (A) and nondirect contact (NDC) (B), over 144 h. The results for 3D traps and hyphal fusions are shown. The corrected values are the differences between the data obtained for fungal strains treated with nematodes and those obtained for fungal strains treated without nematodes.

In the non-direct-contact bioassay, living and dead nematodes were used to evaluate if the fungus had different morphological and metabolic responses. The morphological responses of A. oligospora grown on CMA and PDA to the presence of nematodes under two modes of contact over 144 h were evaluated and found to be significantly different (Fig. 1). When in direct contact with nematodes, the fungal strains grown on both PDA and CMA developed 3D traps. In our study, within 6 h on CMA, the formation of 3D traps was observed, while on PDA the formation of 3D traps was not observed until 12 h. Not only did the fungus grown on CMA produce traps in a shorter time, but also, after 24 h, the fungus grown on CMA had more traps than the fungus grown on PDA. After 30 h, when exposed to nematodes, the fungus cultivated on CMA produced the 3D traps at a level of 100 cm^−2^, while the fungus grown on PDA formed less than half of this number of traps. The fungus grown on PDA took 12 h longer to develop 3D traps at nearly 80 cm^−2^, which is 20% less than that for the fungus grown on CMA (Fig. 1).

The non-direct-contact bioassay was performed according to published protocols (28). The bottom portions of two glass petri plates of identical size were used, with one plate containing the fungal culture and the other containing Caenorhabditis elegans nematodes. The fungal plate was inverted over the plate containing the nematodes, but there was no direct contact between the worms and the fungal mycelium. Under this condition of nondirect exposure to nematodes, at 24 h, no obvious morphological transition was observed in the fungi cultured on either CMA or PDA (Fig. 1). However, hyphal fusions were observed. A morphological transition of hyphal fusions was observed at 30 h for the fungi grown on CMA and at 42 h for the fungi grown on PDA. The fungal strains developed 30% more hyphal fusions when they were grown on CMA than when they were grown on PDA (Fig. 1). Interestingly, the numbers of hyphal fusions produced by the fungus grown on CMA reached the maximum at 80 cm^−2^ within 96 h and then quickly decreased to 30 cm^−2^. However, no such change in the rate of trap formation was observed for the fungal strains grown on PDA. The numbers of hyphal fusions produced by the strains grown on PDA increased steadily and reached 80 cm^−2^ at the end of the observation period. Exposure to living or dead nematodes made no obvious difference in the formation of hyphal fusions when the fungus was grown on CMA. However, on PDA about 20% more hyphal fusions were observed with exposure to live nematodes than with exposure to dead ones (Fig. 1). The formation of 3D traps was not observed for the fungi grown on CMA until 72 h; 3D traps were observed on PDA after 96 h. Then, while the numbers of 3D traps on both media increased slowly, they remained at a low level (Fig. 1). In summary, the fungi grown on CMA developed more traps and did so at a higher rate than those grown on PDA. In the absence of direct contact with nematodes, the fungi grown on either CMA or PDA medium developed more hyphal fusions than 3D traps.

### Metabolites from A. oligospora grown on CMA or PDA during the time course between the saprophytic and pathogenic stages.

The time course metabolite profiles of A. oligospora YMF1.01883 grown on CMA and PDA treated with nematodes, including direct contact and nondirect contact with live and dead nematodes, and treated without nematodes were analyzed by GC-MS analysis (see Tables S1 and S2 in the supplemental material). In the non-direct-contact bioassay, nematodes were also collected at regular intervals for GC-MS analyses in order to remove the effect of the nematodes on A. oligospora in the direct-contact bioassay. Four replicates for each treatment on one medium at one time point led to, in total, 382 fungal samples and 60 nematode samples for metabolite analysis. At one time point, the metabolite profiles of the fungal strains treated with nematodes were compared with those of the fungal strains treated without nematodes to evaluate the metabolites varying in contents. In order to get more information about the potential metabolites, the peaks were designated metabolites if they were identified with a match of 700 on a scale of from 0 to 1,000 to the data for the metabolites in the inborn library. All the metabolites which showed significant changes in concentrations during the time course metabolite profile analyses were considered (Tables S3 to S4).

The metabolite profiles of A. oligospora on CMA and PDA under four treatments at 24 h were analyzed (Fig. S1). It was obvious that despite four types of treatments, the fungal strains on the same medium shared quite similar metabolite patterns. It seemed that direct contact between nematodes and fungi did not make an obvious difference in the fungal metabolite profiles. It is also clear that the metabolite profiles of the fungi grown on CMA were different from those of the fungi grown on PDA. Comparison with the corresponding control groups without nematodes revealed that 34 out of 70 metabolites from the strains on CMA medium and 16 out of 80 metabolites from those on PDA medium were significantly up- or downregulated during the time course profiles of the fungal contacts with nematodes. Among the 34 various metabolites detected from the fungal strains on CMA, 18 metabolites were found from all the three groups treated with nematodes, 13 metabolites were found from the group treated with nematodes in direct contact, 2 metabolites were found from the group treated with live nematodes in nondirect contact, and 1 metabolite was found from the group treated with dead nematodes in nondirect contact. Among the 16 various metabolites detected from the fungal strains on PDA, 9 metabolites were found from all the three groups treated with nematodes, 5 metabolites were found from the group treated with nematodes in direct contact, 1 metabolite was found from the group treated with live nematodes in nondirect contact, and 1 metabolite was found from the group treated with dead nematodes in nondirect contact. These metabolites included short-chain acids and esters, furanones and pyrones, linoleic acid derivatives, purines, and phenolic compounds (Tables S5 and S6).

### Analysis of the metabolic patterns of A. oligospora grown on CMA and PDA between the saprophytic and the pathogenic stages.

**FIG 2:**
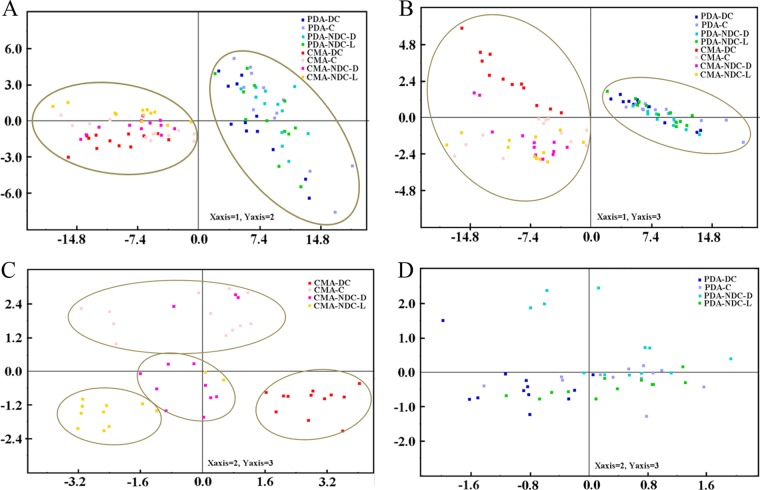
(A) PCA score plots between fungal samples during the saprophytic and pathogenic phases of growth on two different media, CMA and PDA (principal component 1 versus principal component 2; component 1, 0.85; component 2, 0.05); (B) PCA score plots between fungal samples during the saprophytic and pathogenic phases of growth on two different media, CMA and PDA (principal component 1 versus principal component 3; component 1, 0.85; component 2, 0.05; component 3, 0.03); (C) PCA score plot between the saprophytic and pathogenic fungal samples grown on CMA (principal component 2 versus principal component 3; component 1, 0.60; component 2, 0.12; component 3, 0.07); (D) PCA score plot between the saprophytic and pathogenic fungal samples grown on PDA (principal component 2 versus principal component 3; component 1, 0.65; component 2, 0.11; component 3, 0.08). C, A. oligospora growing without nematodes over 144 h as the control group; DC, A. oligospora growing under direct contact with living nematodes over 144 h; NDC-L, A. oligospora growing under nondirect contact with live nematodes over 144 h; NDC-D, A. oligospora growing under nondirect contact with dead nematodes over 144 h.

The profiled metabolite data were analyzed using principal component analysis (PCA). PCA is a useful clustering method for exploratory data analysis and requires no previous knowledge of data structures. The PCA score trajectories of the logarithmically transformed metabolite concentrations from A. oligospora during the saprophytic and the pathogenic stages of growth on CMA and PDA are depicted (Fig. 2). These data points are clustered into two distinct groups in the plot maps (Fig. 2A and B), indicating clear differences in the fungal extract metabolome between fungi growing on the two different media. The first two principal components together accounted for 90.2% of the variance. Overall, the first principal component mainly reflected the differences in the media.

The PCA plots of dendrograms from experiments and metabolite data in CMA and in PDA are depicted in Fig. 2C and D, respectively. The metabolite profiles of the CMA groups displayed more extensive responses to nematodes than those of the PDA groups compared with the responses of their corresponding time series controls. A notable transformation of metabolic changes was observed in the CMA groups treated with nematodes under the two different modes of contact. During the time courses of fungal strains cohabitating with nematodes under two different modes of contact, the PCA plot of the metabolites of fungi grown on CMA shows that the experimental groups separated into four main branches: (i) the control branch of A. oligospora without nematodes (C), (ii) the branch of A. oligospora cohabitating under direct contact with nematodes (DC), (iii) the branch of A. oligospora cohabitating under nondirect contact with live nematodes (NDC-L), and (iv) the branch of A. oligospora cohabitating under nondirect contact with dead nematodes (NDC-D) (Fig. 2C). The fungal strains grown on CMA medium had different metabolic responses not only to the approach of and access to the nematodes but also to the presence of living or dead nematodes. In contrast, on PDA, no obvious distribution of metabolite data in the PCA plot was observed with either the mode of contact or the viability status of the nematodes (Fig. 2D).

**FIG 3:**
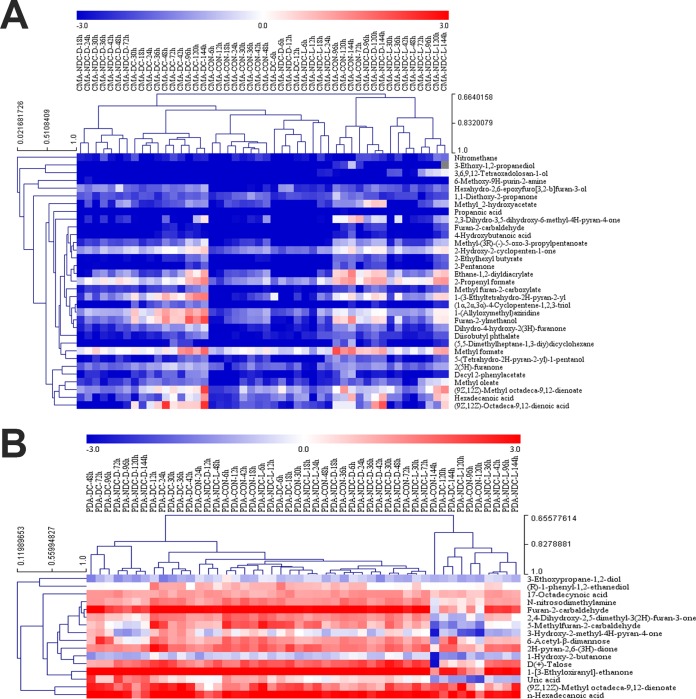
Unsupervised hierarchical clustering of the logarithmically transformed (log_2_) relative concentrations of metabolites from the methanol extracts of A. oligospora YMF1.01883 at different growth phases cultivated in CMA and PDA media. (A) Unsupervised hierarchical clustering of the logarithmically transformed (log_2_) relative concentrations of 33 metabolites from the methanol extracts of wild-type A. oligospora YMF1.01883 cultivated in CMA medium; (B) unsupervised hierarchical clustering of the logarithmically transformed (log_2_) relative concentrations of 16 metabolites from the methanol extracts of wild-type A. oligospora cultivated in PDA medium. C, A. oligospora growing without nematodes over 144 h as the control group; DC, A. oligospora growing under direct contact with nematodes over 144 h; NDC-L, A. oligospora growing under nondirect contact with living nematodes over 144 h; NDC-D, A. oligospora growing under nondirect contact with dead nematodes over 144 h. CON, control.

**FIG 4:**
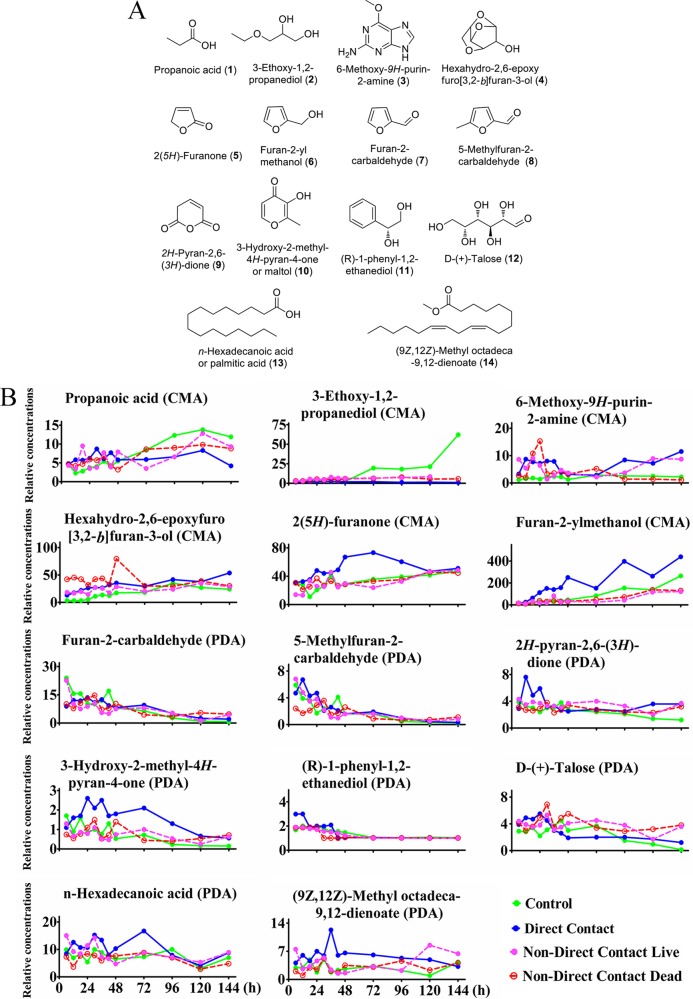
Structures of 14 metabolites (6 metabolites in the CMA group, including metabolites 1 to 6, and 8 metabolites in the PDA group, including metabolites 7 to 14) (A), and their abundances over the time course from the saprophytic to the pathogenic lifestyle of the fungus (B). Green, control group, consisting of A. oligospora fungi growing without nematodes over 144 h; blue, direct contact group, consisting of A. oligospora fungi growing under direct contact with living nematodes over 144 h; rose, non-direct-contact live group, consisting of A. oligospora fungi growing under nondirect contact with living nematodes over 144 h; red, non-direct-contact dead group, consisting of A. oligospora fungi growing under nondirect contact with dead nematodes over 144 h (*n* = 4).

Hierarchical clustering was applied to organize the metabolites on the basis of their relative levels across samples and to discern linkages between these metabolites (Fig. 3). A subset of small molecular metabolite categories, including 11 metabolites in the CMA group and 9 metabolites in the PDA group, was significantly changed while the fungi cohabited with nematodes from 6 h to 96 h in both media. Among these metabolites, 6 metabolites in the CMA group consistently changed patterns during the time course. These were propanoic acid (compound 1), 3-ethoxy-1,2-propanediol (compound 2), 6-methoxy-9*H*-purin-2-amine (compound 3), hexahydro-2,6-epoxyfuro[3,2-*b*]-3-ol (compound 4), 2(5*H*)-furanone (compound 5), and furan-2-ylmethanol (compound 6) (Fig. 4). On PDA medium, 7 metabolites showed changing patterns during the time course metabolite profiles. These were furan-2-carbaldehyde (compound 7), 5-methylfuran-2-carbaldehyde (compound 8), *2H*-pyran-2,6(3*H*)-dione (compound 9), 3-hydroxy-2-methyl-4*H*-pyran-4-one (compound 10), (*R*)-1-phenyl-1,2-ethanediol (compound 11), d-(+)-talose (compound 12), *n*-hexadecanoic acid (compound 13), and (9*Z*,12*Z*)-methyl octadeca-9,12-dienoate (compound 14; the methyl ester of linoleic acid) (Fig. 4). These compounds may have potential functional roles in the interaction between the fungal strains and nematodes.

### Characterization of target metabolites during the fungus-nematode interaction.

The roles of 12 of the 14 individual metabolites were evaluated using 12 commercially available compounds, including propanoic acid (compound 1), 3-ethoxy-1,2-propanediol (compound 2), 6-methoxy-9*H*-purin-2-amine (compound 3), 2(5*H*)-furanone (compound 5), furan-2-ylmethanol (compound 6), furan-2-carbaldehyde (compound 7), 5-methylfuran-2-carbaldehyde (compound 8), 3-hydroxy-2-methyl-4*H*-pyran-4-one (compound 10), (*R*)-1-phenyl-1,2-ethanediol (compound 11), d-(+)-talose (compound 12), *n*-hexadecanoic acid (compound 13), and (9*Z*,12*Z*)-methyl octadeca-9,12-dienoate (compound 14). The use of chemical standards allowed us to test the ability of individual compounds to attract or poison nematodes, as well as to observe their effects on fungal development and morphology.

**FIG 5:**
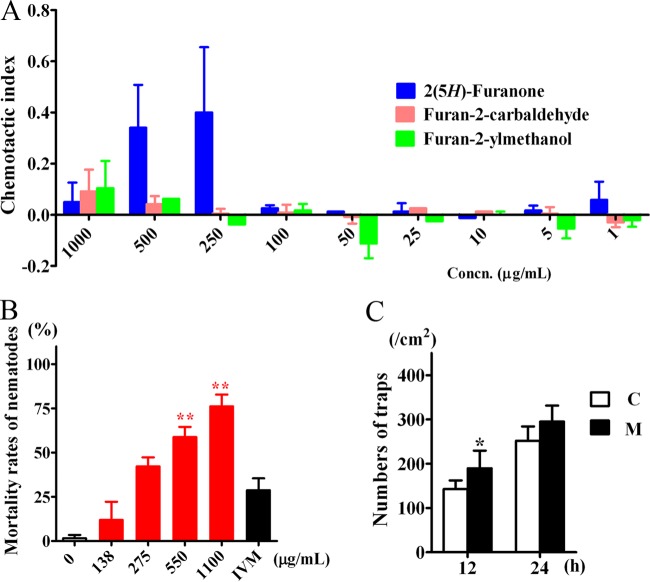
(A) Attracting activities of three metabolites, 2(5*H*)-furanone (compound 5), furan-2-ylmethanol (compound 6), and furan-2-carbaldehyde (compound 7), for the nematode C. elegans. (B) Effect of 5-methylfuran-2-carbaldehyde (compound 8) on the mortality of C. elegans at 12 h, with 1 μg/ml ivermectin (IVM) used as a positive control. (C) Effect of maltol (compound 10; M) at a concentration of 2.5 μg/ml on the trap formation of A. oligospora treated without maltol as a control (C). **, *P* < 0.01; *, *P* < 0.05 (*n* = 5).

During the preliminary chemotaxis bioassay, among the metabolites tested at concentrations of 1 mg/ml, 0.1 mg/ml, and 0.01 mg/ml, C. elegans worms were attracted toward only three metabolites: 2(5*H*)-furanone (compound 5), furan-2-yl methanol (compound 6), and furan-2-carbaldehyde (compound 7). Interestingly, all three of these metabolites share a furan ring and have similar molecular weights. Compounds 5 and 6 were characterized from the fungus on CMA, while compound 7 was characterized from the fungus on PDA, based on the time course metabolite profiles of the fungal strains (Fig. 4). To characterize the chemotaxis responses to these three volatile attractants further, worms were tested with these compounds at concentrations of 1,000, 500, 250, 100, 50, 25, 10, 5, and 1 μg/ml, and a chemotaxis index was calculated on the basis of the enrichment of animals at the attractant. The chemotaxis index could vary from 1.0 (perfect attraction) to −1.0 (perfect repulsion). The weakly attractive compound ethanol was used as the control. The metabolite 2(5*H*)-furanone (compound 5) functioned as an attractant through a broad range of concentrations, displaying the strongest nematode-attracting ability at a concentration of 250 μg/ml (Fig. 5A). Furan-2-yl methanol (compound 6) showed a more complex response, being attractive when undiluted but somewhat repulsive at low concentrations.

Among the 12 metabolites tested for their toxicity toward C. elegans, 5-methylfuran-2-carbaldehyde (compound 8) showed toxic activity against the nematodes with a 50% lethal concentration (LC_50_) of 369 μg/ml in 12 h. The other compounds tested did not display obvious toxic effects at the concentrations tested in these experiments (Fig. 5B).

The same 12 compounds were applied to the fungal cultivation media. In comparison with the findings obtained with the solvent control, fungal strains treated with 2.5 μg/ml of 3-hydroxy-2-methyl-4*H*-pyran-4-one (compound 10), also known as maltol (compound 10), displayed a significant increase in the formation of 3D traps induced by nematodes. Over 12 h, the number of adhesive 3D traps formed by the fungus grown on the media treated with maltol (compound 10) was 189 cm^−2^ (Fig. 5C), or 30% more than that on control untreated media (142 cm^−2^).

### Functional validation of the furanone and pyrone metabolites during the fungus-nematode interaction.

**FIG 6:**
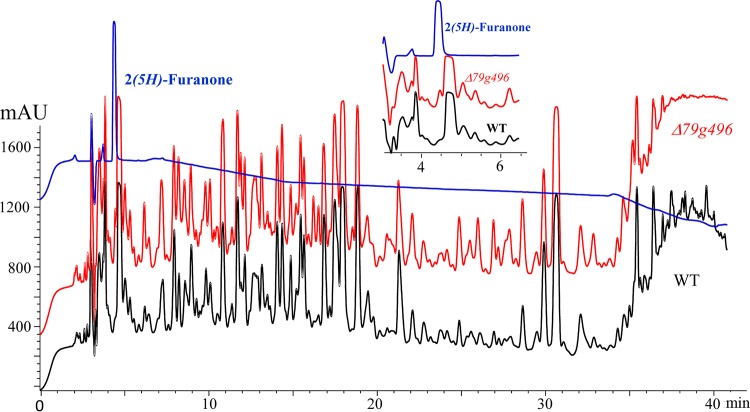
HPLC analysis of the methanol extracts of the PDA culture broths from the wild-type strain (black line) and the Δ*AOL_s00079g496* (Δ*79g496*) mutant (red line). Blue lines, the peak of 2(5*H*)-furanone (compound 5) at about 4 min. mAU, milli-absorbance units.

**FIG 7:**
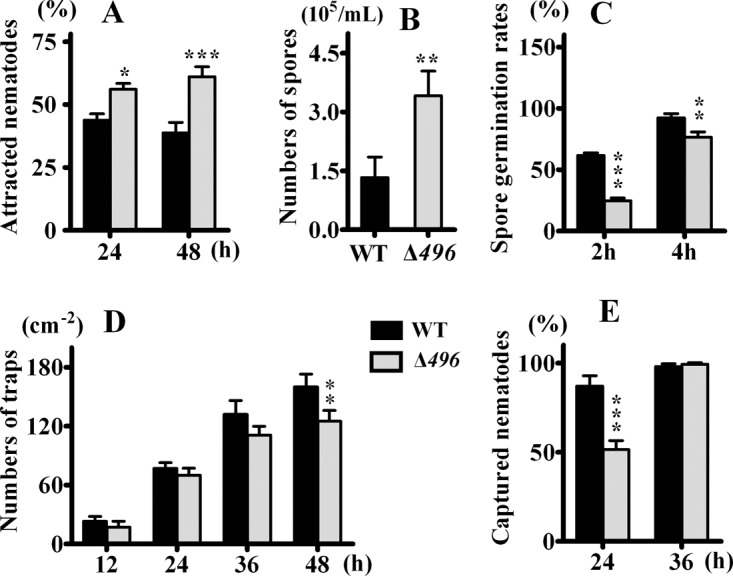
(A) Comparison of attracting activities of wild-type (WT) A. oligospora and the Δ*AOL_s00079g496* (Δ*496*) mutant; (B) comparison of spore formations of wild-type *A*. oligospora and the Δ*AOL_s00079g496* mutant; (C) comparison of spore germination rates of wild-type A. oligospora and the Δ*AOL_s00079g496* mutant; (D) comparison of trap formation of wild-type A. oligospora and the Δ*AOL_s00079g496* mutant; (E) comparison of nematode-capturing abilities of wild-type A. oligospora and the Δ*AOL_s00079g496* mutant. ***, *P* < 0.001; **, *P* < 0.01; *, *P* < 0.05 (*n* = 4).

Bioinformatics analysis of the A. oligospora genome revealed five putative polyketide synthase (PKS) genes, including a type III PKS synthase gene, *AOL_s00043g287* (*PKS III-1*), and four type I PKS synthase genes, *AOL_s00043g828* (*PKS I-1*), *AOL_s00079g496* (*PKS I-2*), *AOL_s00215g283* (*PKS I-3*), and *AOL_s00215g926* (*PKS I-4*) (29). Disruption of these five PKS genes was performed, and five mutants, the Δ*AOL_s00043g287* (*PKS III-1*), Δ*AOL_s00043g828* (*PKS I-1*), Δ*AOL_s00079g496* (*PKS I-2*), Δ*AOL_s00215g283* (*PKS I-3*), and Δ*AOL_s00215g926* (*PKS I-4*) mutants, from 20, 7, 8, 22, and 23 transformants, respectively, were screened by genomic DNA isolation and diagnostic PCR (Fig. S2 and S3). The metabolites from cultures of these mutants and the wild-type strain were extracted and analyzed by high-performance liquid chromatography (HPLC) and GC-MS methods according to standard protocols (29). In the HPLC profiles, the Δ*AOL_s00215g283* (*PKS I-3*) and Δ*AOL_s00215g926* (*PKS I-4*) mutants lacked most of the peaks, with retention times ranging from 21 to 40 min (Table S8). The Δ*AOL_s00043g287* (*PKS III-1*) mutant displayed the same HPLC and GC-MS profiles as the wild-type strain. The Δ*AOL_s00043g828* (*PKS I-1*) mutant showed three peaks in the HPLC profile at retention times of 11.68, 17.87, and18.82 min that were not observed in the wild type and that were then characterized as nonfuranone and nonpyrone metabolites by comparison with the standard samples and further GC-MS analysis. Only the HPLC profile of the Δ*AOL_s00079g496* (*PKS I-2*) mutant displayed an obvious difference from the profile of the wild type, which was found in the peak for the attractant compound 2(5*H*)-furanone (compound 5), while most of the other peaks were similar to those in the wild-type profile (Fig. 6). It was interesting to note that even when it was grown on PDA, the Δ*AOL_s00079g496* (*PKS I-2*) mutant yielded a level of 2(5*H*)-furanone (compound 5) 200% higher than that yielded by the wild-type strain (Fig. 6). Further chemotaxis bioassays performed on CMA also revealed that 150% more worms were attracted to the Δ*AOL_s00079g496* (*PKS I-2*) mutant than to the wild-type strain (Fig. 7), strongly confirming the nematode-attracting function of 2(5*H*)-furanone (compound 5).

**FIG 8:**
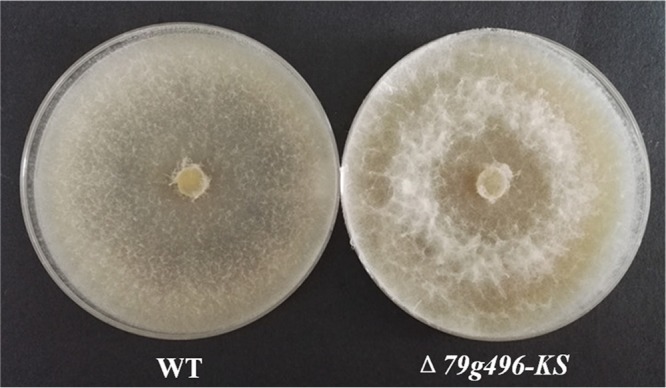
Comparison of the mycelial morphology of the wild-type strain and the Δ*AOL_s00079g496* mutant (Δ*79g496-KS*) on PDA plates (15 days).

**FIG 9:**
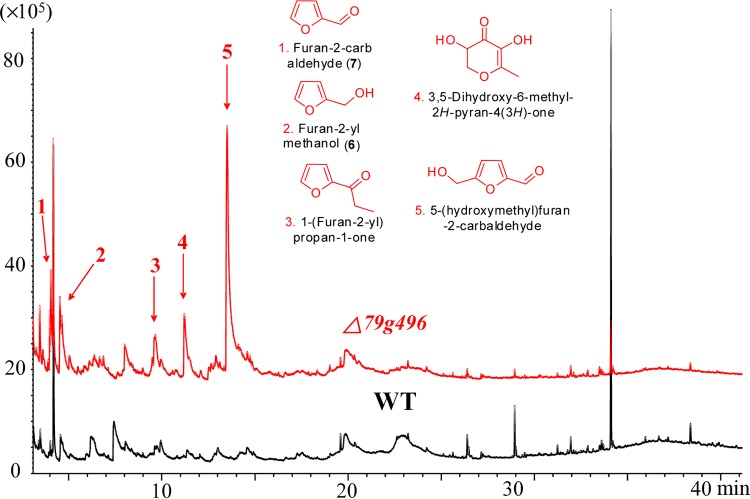
GC-MS analysis of the methanol extracts of the wild-type strain (black line) and the Δ*AOL_s00079g496* (Δ*79g496*) mutant (red line) on CMA. Red arrows, compounds detected in the mutant strain.

The Δ*AOL_s00079g496* (*PKS I-2*) mutant showed the same growth rates and morphological characteristics as the wild-type strain both on CMA and on PDA within 6 days. However, from 9 days on, the Δ*AOL_s00079g496* mutant grown on PDA displayed much fluffier aerial mycelia than the wild type (Fig. 8). In addition, the Δ*AOL_s00079g496* mutant showed increased spore formations but decreased germination rates compared with those for the wild-type strain (Fig. 7). When nematodes were added to 4-day-old PDA cultures, it was surprising to note that the Δ*AOL_s00079g496* mutant formed fewer traps than the wild-type strain (Fig. 7). After 24 h, the number of adhesive traps formed by the Δ*AOL_s00079g496* mutant was 69 cm^−2^, which was 10% less than the number formed by the wild-type strain (77 cm^−2^) (Fig. 7). Accordingly, the number of nematodes infected by the traps produced by the Δ*AOL_s00079g496* mutant was 35% less than that for the wild-type strain (Fig. 7). Nevertheless, in both the wild-type and the mutant strains, all the nematodes were dead within 36 h, despite the fact that the Δ*AOL_s00079g496* mutant made fewer traps. Further GC-MS analysis revealed that the Δ*AOL_s00079g496* (*PKS I-2*) mutant produced more furanone and pyrone metabolites, including furan-2-ylmethanol (compound 6), furan-2-carbaldehyde (compound 7), 1-(furan-2-yl)propan-1-one (an ethyl derivative of compound **7**), 5-(hydroxymethyl)furan-2-carbaldehyde (an hydroxy derivative of nematicidal compound 8), and 3,5-dihydroxy-6-methyl-*2H*-pyran-4(*3H*)-one (an hydroxy derivative of compound 10), than the wild-type strain (Fig. 9).

## DISCUSSION

Nematode-trapping fungi have fascinated scientists for decades, and many earlier workers have observed the way in which the presence of nematodes alters the morphology and metabolism of trap-forming species. Although earlier studies detected attractant and nematocidal metabolites by their activities, the compounds were never chemically identified (21–27, 30). Therefore, we hypothesized that these signaling molecules might be volatile in nature. In our analyses, we used GC-MS and were able to separate and chemically characterize the metabolites, as well as elucidate their biological activities in attracting nematodes, in inducing trap formation, or in killing nematodes.

Under direct physical contact with nematodes, fungi grown on CMA produced more 3D traps than those grown on PDA and did so at a higher rate. Similar results were obtained in the non-direct-contact bioassay; however, instead of 3D trap formation, fungal hyphal fusions were observed in the nondirect assay. The fungi grown on CMA developed more hyphal fusions and 3D traps than those grown on PDA. Only at a late stage, after 72 h on CMA and 96 h on PDA, were a few 3D traps observed. Previous studies suggested that trap formation also requires a hyphal fusion event during initial stages (31), and hyphal fusions were regarded as defensive structures of nematode-trapping fungi (5, 26). This might indicate that in the face of the approaching nematodes, the fungus first moves into a defensive posture through hyphal fusion before trap formation is induced by direct contact with nematodes. Both bioassays revealed that A. oligospora displayed greater morphological transitions in response to the presence of nematodes when it was grown on CMA than when it was grown on PDA.

The time course of metabolite profiling indicated that the growth medium influenced metabolism more profoundly than the mode of contact with nematodes. When grown on CMA, almost half (48%) of the total detected metabolites changed in abundance in response to physical or indirect contact with nematodes; when grown on PDA, only 11% of the metabolites displayed significant changes in abundance in response to direct or indirect contact with nematodes. The fungal strains grown on CMA medium had more extensive metabolic responses to nematodes than those grown on PDA medium. The PCA plot of metabolite data from fungal strains grown on CMA clustered into four main branches, corresponding to the experimental treatments according to the mode of contact (direct or indirect) and the status of the nematodes (living or dead). In other words, the fungal strains grown on CMA had different metabolic patterns in response not only to the approach of nematodes but also to the presence of living or dead nematodes. In summary, our morphological and metabolic analyses indicate complex relationships between media, fungal sensitivity, and morphological transitions. When A. oligospora was grown on CMA, it made quicker and stronger responses in both morphology and metabolism even before having direct contact with the nematodes.

The metabolomics analyses suggest a role of particular volatile metabolites in initiating the morphological transition of the nematode-trapping fungus. Volatile compounds are emitted by many species of fungi and serve many ecological functions in nature, and they have also been exploited for their role in food flavor and as indirect indicators of the presence of fungal growth (32–34). However, the role of individual fungal volatile substances in fungus-nematode ecological interactions is poorly understood.

Metabolites 1 to 14 were screened out from the metabolite profiles of the fungal strains grown on CMA and PDA during the switch from the saprophytic to the pathogenic stage. Among them, two furanone metabolites (metabolites 5 and 6) emitted by the fungus grown on CMA were found to attract the nematodes to the fungal colony. An early study on comparison of the interaction of the free-living nematode Panagrellus redivivus with nematophagous fungi and nonnematophagous fungi showed that nematophagous fungi preferred to attract nematodes, while nonnematophagous fungi repelled them (30). Our data not only confirm that the nematode-trapping fungi can chemically lure the prey to traps but also identify specific attractive compounds to be volatile in nature.

Among the metabolites produced by the fungus grown on PDA that changed between the saprophytic and pathogenic phases, one volatile furanone metabolite, 5-methylfuran-2-carbaldehyde (compound 8), was found to significantly paralyze and kill the nematodes. There is a long-standing assumption that an unstable nematode-inactivating chemical compound produced by the fungus might be a volatile metabolite (21), and our work confirms this assumption. We also found that the amount of the long-chain metabolite (9*Z*,12*Z*)-methyl octadeca-9,12-dienoate (compound 14; the methyl ester of linoleic acid) increased significantly in the fungus grown on PDA under contact with nematodes. Other research had identified linoleic acid to be a nematicidal metabolite in the mycelial extracts of several pathogenic fungi of the genus Arthrobotrys (22). However, in our study, linoleic acid and its ethyl ester did not show obvious inhibitory effects on nematodes.

In addition to the furanone metabolites involved in the interaction between the fungus and the nematodes, a volatile pyrone metabolite, 3-hydroxy-2-methyl-4*H*-pyran-4-one (maltol; compound 10), was identified to be a morphological regulator. Maltol (compound 10) is found widely in various beans and other plant sources, such as larch tree bark, pine needles, and roasted malt (from which it gets its name) (35), but it has rarely been described as a microbial metabolite (36, 37). Maltol is responsible for much of the characteristic smell of red ginseng (38) and has been used to impart a sweet aroma to commercial fragrances. Maltol has been marketed as a safe and reliable food flavor-enhancing agent for freshly baked breads and cakes and also as a food preservative and natural antioxidant (39). Maltol is also used as a bidentate metal ligand for administered drugs (37). A recent study revealed that the maltol found in root exudates from crabgrass can affect the growth of maize shoots and reduce the soil microbial biomass carbon by acting as an allelochemical that interferes with plant growth and the microbial community of soils (40). However, our study indicates that maltol acts as a morphological regulator for fungi. In our study, maltol was found to be involved in regulating the formation of 3D traps by the nematode-trapping fungus. Since maltol is widely distributed in plants, it is interesting to speculate whether there is a coevolutionary relationship between the maltol from plants attacked by the nematodes and the induction of trap formation by nematode-trapping fungi.

Furanone and pyrone metabolites are known to be important plant fruit constituents (41, 42). For example, the 4-hydroxy-3(2*H*)-furanones associated with fruit aromas act to attract animals to fruits in order to ensure seed dispersal. Furanones may function as interorganism signal molecules in various plant ecosystems (43). In plants, furanone and pyrone metabolites originate directly from carbohydrate hexoses and pentoses as Maillard reaction products (44). Previous studies have suggested that furanone might be derived from phosphorylated carbohydrates in tomato and the yeast *Saccharomyces rouxii* and that furaneol was derived from d-fructose-1,6-diphosphate. Hexose diphosphate was also assumed to be a biogenetic precursor to 4-hydroxy-5-methyl-2-methylene-3(2*H*)-furanone, likely converted by an as yet unknown enzyme in tomato (Solanum lycopersicum) and strawberry (Fragaria ananassa) (35, 37). However, the biogenetic pathways of furanones and those of pyrones, such as maltol (compound 10), remain unknown.

Polyketides are the most abundant class of fungal secondary metabolites (16). Because polyketides, furanones, and pyrone metabolites all derive from the same precursors that are obtained from hexose utilization, we studied the effects of all the PKS genes on the production of furanones and pyrone metabolites in A. oligospora. Our previous report revealed that the knockout of the PKS I-3 gene (*AOL_s00215g283*) led to the abolishment of the morphological regulatory arthrosporols and high levels of trap formation (29). To elucidate the effects of genes on the production of furanone and pyrone metabolites, mutants deficient in each of the five PKS genes of A. oligospora were constructed. The mutant that lost the PKS I-2 gene (*AOL_s00079g496*) showed increased production of the furanone and pyrone metabolites on both CMA medium and PDA medium, with no obvious changes in other metabolites or in its morphology. The content of nematode-attracting furanone and pyrone metabolites in the mutant strain was double that in the wild-type strain. The fact that the Δ*AOL_s00079g496* (*PKS I-2*) mutant displayed 150% stronger nematode-attracting activity was in good agreement with the 200% higher levels of attractants in the mutant strain. Although there were fewer traps induced by the strain in which the PKS I-2 gene (*AOL_s00079g496*) had been knocked out, the overall predatory ability remained the same as that in the wild type. We hypothesize that the extra production of nematode-toxic furanone metabolites compensated for the lower number of traps.

**FIG 10:**
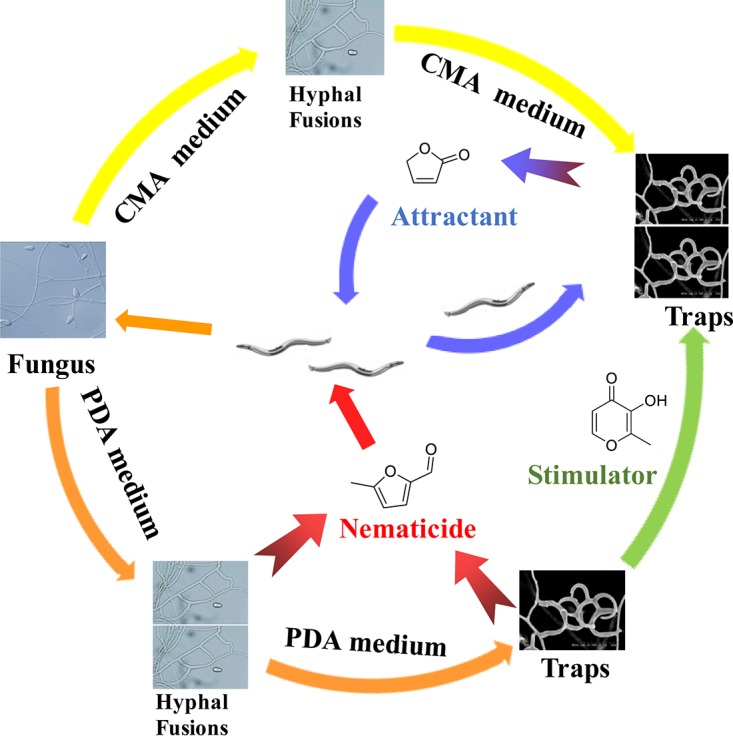
Morphological and metabolic adaptation of wild-type A. oligospora on two media, CMA and PDA. In direct contact with nematodes, the fungus grown on CMA develops more traps than that grown on PDA; in nondirect contact with nematodes, the fungus grown on PDA develops more hyphal fusion than that grown on CMA. The fungus grown on CMA produced an attractant molecule, 2(5*H*)-furanone, while the fungus grown on PDA produced a nematicide metabolite, 5-methylfuran 2-carbaldehyde, as well as a stimulator, maltol, that increased trap numbers.

In conclusion, we found that A. oligospora grown on CMA and PDA can sense approaching nematodes and develop hyphal fusions (Fig. 10). A. oligospora grown on both CMA and PDA produced small volatile furanone and pyrone metabolites in response to the presence of nematodes. The fungus cultivated on CMA medium made furanone metabolites that attracted nematodes, while the fungus grown on PDA medium produced nematode-toxic furanone metabolites (Fig. 10). A mutation resulting in increases in the amounts of furanone and pyrone metabolites led to increased attractive activity and decreased trap formations of the A. oligospora mutants, confirming the results presented above from the integrated morphological and metabolic analysis. These results show that the fungus flexibly adjusts its metabolic activity to complement morphological changes, thereby potentially affecting fungal nematode-trapping ability and differential trap formation.

## MATERIALS AND METHODS

### Fungal and nematode strains, media, and treatment conditions.

A. oligospora model species strain YMF1.01883 (ATCC 24927) was used in the fungus-nematode interaction bioassays and cultured on CMA (corn [Kunming, China], 20 g liter^−1^; agar [Biofroxx, Einhausen, Germany], 15 g liter^−1^) or PDA (potato [Kunming, China], 200 g liter^−1^; glucose [Solarbio, Beijing, China], 10 g liter^−1^; agar, 15 g liter^−1^). All the bioassays were conducted in 9-cm-diameter glass petri dishes. Inocula of A. oligospora YMF1.01883 were cultured at 28°C on PDA plates for 1 week. Then, one 5-mm-diameter disk of the fungus was cut with a sterile cork borer and was inoculated onto either CMA or PDA. To obtain strong fresh mycelia, the cultures were incubated at 28°C until the fungal lawn occupied half of the petri plate.

Two treatment bioassays were performed to evaluate the fungal responses during the A. oligospora-nematode interaction: direct physical contact and nondirect contact. Caenorhabditis elegans (strain N2) was cultured in oatmeal medium at 22°C for 6 to 7 days. The fungal strains treated without nematodes on PDA or CMA were used as controls for both treatment bioassays.

For the non-direct-contact bioassay, the bottom of the petri plate containing the fungal lawn was inverted over a second petri plate bottom of identical size containing 1 ml solution of mixed-stage living nematodes or dead nematodes (28). In this treatment, the fungi and the nematodes shared the same atmosphere but had no direct physical contact. The two halves of the petri plates were sealed together with Parafilm and then incubated in a dark chamber at 28°C. For the control group, 1 ml sterile H_2_O was used in place of the nematode suspension. Half of the live nematodes were submerged for 20 min in 45°C water to prepare dead nematodes. In each treatment, the fungus and nematodes were harvested separately at 6-h intervals for the first 48 h and at 24-h intervals for the subsequent 4 days. In total, four biological replicates were performed for metabolomic analysis.

For the direct-contact bioassay, 1 ml mixed-stage living nematode solution (about 3,000 nematodes) was directly added in the center of the fungal lawn; 1 ml sterile H_2_O was used as a control. The petri dishes were sealed with Parafilm and incubated in a dark chamber at 28°C. The fungal lawn was observed and harvested at 6-h intervals for the first 48 h and at 24-h intervals for the subsequent 4 days. The fungal lawns with nematodes from each time point were extracted with methanol for metabolomic analysis.

### Morphological analysis.

For characterization of fungal growth, development, and morphological transitions, microscopy was performed according to the protocols outlined previously (16). The morphological transitions of the hyphal fusions to two-dimensional (2D) nets and morphological transitions to three-dimensional (3D) traps were assessed at 6-h intervals for the first 48 h and at 24-h intervals for the subsequent 4 days. The hyphal fusions and 3D traps were evaluated with a binocular microscope (magnification, ×10; Olympus, Japan). Seven fields in each fungal culture were picked at random for observation, microscopic counting was repeated three times, and the data obtained were analyzed statistically. Image stacks were processed using Imaris (version 6.3.1) software (Bitplane) to generate images for publication. The mean corrected data for the fungal strains treated with nematodes were obtained from the outcome of the data for the test group minus the data for the control group.

### Metabolomic profiling.

The metabolomic profiling analysis involved sample extraction, metabolite detection, metabolomic data preprocessing (e.g., metabolite feature extraction, chromatographic peak alignment, data reduction), and statistical analysis. The metabolite profiles were obtained from the direct-contact and non-direct-contact bioassays conducted on the two media. Each treatment group consisted of 4 replicates and a corresponding control group with the same number of replicates. The fungal mycelial lawn was harvested and extracted twice with 30 ml methanol under ultrasonic conditions for 30 min in an ice-cooled bath-type sonicator. Each methanol-soluble extract was centrifuged for 3 min at 10,000 × *g* and 4°C, and the supernatant was concentrated to dryness under vacuum. Each dried extract was resuspended in 1 ml methanol under ultrasonic conditions for 20 min in an ice-cooled bath-type sonicator and then filtered through 0.22-μm-pore-size membranes. The filtrates were stored at −80°C prior to GC-MS analyses.

GC-electrospray ionization (EI)-MS analyses were performed as described previously (29) using a Hewlett-Packard 5890 series II Plus gas chromatograph linked to a Hewlett-Packard 5972 mass spectrometer system (Hewlett-Packard, San Diego, CA, USA) equipped with a 30-m-long and 0.25-mm (inside diameter) HP5-MS capillary column with a 0.5-μm film thickness. The temperatures were programmed from 100 to 300°C at a rate of 5°C/min. Helium was used as a carrier gas at a flow rate of 0.7 ml/min. The split ratio was 1:20, the injector temperature was 280°C, the interface temperature was 300°C, and the ionization voltage was 70 eV.

Identification of peaks was performed through determination of the retention time index and mass spectrum. Compounds from the strains were designated metabolites if they were identified with a match of 900 on a scale of from 0 to 1,000 and a retention index (RI) deviation of 3.0 (45, 46). The semiquantitative analysis of the main compounds was performed through internal normalization to the area of each compound. The addition of each area of the compounds corresponds to a 100% area (47).

### Data analysis.

The data matrix was analyzed by principal component analysis (PCA) (48). The principal component calculations were performed using the J. Craig Venter Institute MultiExperiment Viewer (MeV) software with a centering mode, based on means, and visualized by using the eigenvalues of the first principal component (*x* axis) and the second principal component (*y* axis) or the second principal component (*x* axis) and the third principal component (*y* axis) (48). Each point on the plot represents an individual sample, and each point on the loading plot represents a contribution of an individual metabolite to the score plot. Accordingly, chemical components responsible for the differences between samples detected in the score plot can be extracted from the corresponding loadings.

Samples were clustered using unsupervised hierarchical cluster analysis (HCA), which provides an organization of primary data sets without a predefined classification. Data were visualized by the use of dendrograms. Logarithmic values of metabolite relative concentrations were implemented in the J. Craig Venter Institute MeV software in an unsupervised HCA using the Pearson correlation (48, 49).

### Chemotaxis and nematode-toxic assays.

In order to evaluate if these volatile metabolites and mutants have nematode-attracting ability, chemotaxis assays were performed in 9-cm plates containing assay medium (20% agar, 5 mM potassium phosphate, pH 6.0, 1 mM CaCl_2_, 1 mM MgSO_4_) according to published protocols (50, 51). Two marks at opposite ends were made on the back of the petri plate about 1 cm from the edge of the plate. Between 100 and 200 washed adult nematodes were placed near the center of a 9-cm assay plate, with the putative attractant being placed over the mark at one end of the plate and an aliquot of 1 μl the solvent ethanol being placed over the other mark as a control. An aliquot containing each respective test compound was suspended in 1 μl of ethanol and placed on the agar over one mark. Test compounds, including propanoic acid (compound 1), 3-ethoxy-1,2-propanediol (compound 2), 2(5*H*)-furanone (compound 5), furan-2-ylmethanol (compound 6), furan-2-carbaldehyde (compound 7), 5-methylfuran-2-carbaldehyde (compound 8), *n*-hexadecanoic acid (compound 13), and (9*Z*,12*Z*)-methyl octadeca-9,12-dienoate (compound 14), were obtained from Sigma-Aldrich, USA, and d-(+)-talose (compound 12) was obtained from TCI Tokyo Chemical Industry Co., Ltd. Japan. To evaluate the mutant strains, a 6-mm-diameter disk of mycelium grown for 2 days on CMA medium was used as a test sample. For the negative controls, a 6-mm-diameter disk of the wild-type strain on CMA medium was used. About 100 washed C. elegans adult nematodes in M9 buffer were placed near the center of the plate, equidistant from the two marks. After 1 h, the number of C. elegans nematodes at the putative attractant area and at the control area was counted. A chemotaxis index with the following formula was calculated on the basis of the enrichment of animals at the attractant: chemotaxis index = (number of nematodes at the attractant area − number of nematodes at the control area)/total number of nematodes. The chemotaxis index varied from +1.0 to −1.0. In this assay, a chemotaxis index of 1.0 represents a complete preference for the test sample, and an index of 0 represents an equal distribution.

The nematode toxicity test was performed according to a previously published protocol (52). About 300 C. elegans nematodes were dispensed into 3.5-cm plates containing 1 ml of M9 buffer with variable amounts of pure metabolites (dissolved in dimethyl sulfoxide [DMSO]) per plate. The same volume solvent of DMSO (0.5% [vol/vol] DMSO) was used as a negative-control group, and 1 μg/ml ivermectin (Sigma-Aldrich, USA) was used as a positive control. The worms were exposed for 24 h at 20°C, and the number of dead or living worms was determined by the absence/presence of touch-provoked movement when probed with a platinum wire. The median lethal concentration (LC_50_) value was calculated using the probit method (52). All treatments were conducted in triplicate.

### Mutant construction.

The annotation of the genome of A. oligospora revealed five putative PKS genes, including *AOL_s00043g287*, *AOL_s00043g828*, *AOL_s00079g496*, *AOL_s00215g283*, and *AOL_s00215g926* (16). The gene *AOL_s00043g287* encodes a type III PKS and is designated *PKS III-1*. The genes *AOL_s00043g828*, *AOL_s00079g496*, *AOL_s00215g283*, and *AOL_s00215g926* encode type I PKSs and are designated *PKS I-1*, *PKS I-2*, *PKS I-3*, and *PKS I-4*, respectively. A modified protoplast transformation method (29) for genetic disruption of these PKS genes was applied using double-crossover recombination with the hygromycin resistance (Hyg^r^) gene as a selection marker, followed by identification of the desired mutants using diagnostic PCR. The two homologous regions were amplified from A. oligospora genomic DNA using primers containing overlapping regions with the vector pAg1-H3 and the Hyg resistance cassette.

Genomic DNA of A. oligospora was extracted as previously described (16). Restriction endonucleases and DNA-modifying enzymes were purchased from New England BioLabs (Beverly, MA). In-Fusion HD cloning kits were purchased from Clontech Laboratories (Mountain View, CA). The left and right DNA fragments flanking the hygromycin resistance (Hyg^r^) gene in the pAg1-H3 vector were amplified from the genomic DNA of A. oligospora by PCR (GXL high-fidelity DNA polymerase [TaKaRa Biotechnology Co. Ltd., Dalian, China]) using the primer sets described below. The disruption vector for PKS III-1 gene (*AOL_s00043g287*) was constructed with primer set 287-5f (TCGAGCTCGGTACCAAGGCCCGGGTAAGACGGTGTAGAGGGCTGC) and 287-5r (GAGGCCTGATCATCGATGGGCCCGGACTTAGACTGGGCACT) and primer set 287-3f (GCGATCGCGGCCGGCCGGCGCGCCGCCGAGGTCTTCTGGAAA) and 287-3r (GAGTCACGAAGCTTGCATGCCTGCAGGTGTGCCGTTGCTTGGTAA). The disruption vector for the PKS I-1 gene (*AOL_s00043g828*) was constructed with primer set 828-5f (GAGCTCGGTACCAAGGCCCGGGTGCGTCACTTTGTTCATC) and 828-5r (CGAGGCCTGATCATCGATGGGCCCTAAATCTATCGTCGGGTAC) and primer set 828-3f (GCGATCGCGGCCGGCCGGCGCGCCTCACGGAACAGGCACTAC) and 828-3r (TCACGAAGCTTGCATGCCTGCAGGCAGACGATCTATCCCACC). The disruption vector for the PKS I-2 gene (*AOL_s00079g496*) was constructed with primer set 496-5f (AGCTCGGTACCAAGGCCCGGGTTTGTTATAGAAATGCCTCC) and 496-5r (GAGGCCTGATCATCGATGGGCCCGTCTTACCCAACTTAGCG) and primer set 496-3f (GCGATCGCGGCCGGCCGGCGCGCCAGATAGTAAGGATGGGCAG) and 496-3r (TCACGAAGCTTGCATGCCTGCAGGTGAAACGCAGACGGGTAA). The disruption vector for the PKS I-3 gene (*AOL_s00215g283*) was constructed with primer set 283-5f and 283-5r and primer set 283-3f and 283-3r (29). The disruption vector for the PKS I-4 gene (*AOL_s00215g926*) was constructed with primer set 926-5f (GAGCTCGGTACCAAGGCCCGGGGCCGTAAGTAAATTGTCTG) and 926-5r (AGGCCTGATCATCGATGGGCCCCAAGTGCGTGGTAGGAGC) and primer set 926-3f (TCTAGAGGATCCCCCGACTAGTGTGGCGTTCGTAGTGATG) and 926-3r (CACGAAGCTTGCATGCCTGCAGGTTCCAGTAGGACCGTGTA).

The DNA fragments (5′ flanks and 3′ flanks) were purified using a PCR cleanup kit (Macherey-Nagel Inc., Düren, Germany) and NucleoSpin gel and were inserted into the specific sites of the pAg1-H3 vector by the In-Fusion method to generate the completed disruption of the pAg1-H3-5′-3′ vector. Amplifications of the homologous fragments were carried out as follows. An amplification system with a 25-μl PCR mixture with GXL high-fidelity DNA polymerase was applied following the manufacturer's instructions (TaKaRa). Genomic DNA prepared from A. oligospora (0.5 μl) was added as the template. All PCRs were performed in a Veriti 96-well thermal cycler (Applied Biosystems, Foster City, CA). The amplification program consisted of predenaturation at 98°C for 4 min, followed by 30 cycles of denaturation at 98°C for 10 s, annealing at 57°C for 15 s, and elongation at 68°C for 2 min, with a final extension step at 68°C for 10 min.

PDASS medium (PDA supplemented with 0.6 M sucrose, 0.3 g/liter yeast extract, 0.3 g/liter tryptone, 0.3 g/liter peptone, and 200 μg/ml hygromycin B [Roche Applied Science, Mannheim, Germany] for the selection of transformants) was applied to carry out protoplast regeneration. Four 1- to 1.2-cm diameter mycelial plugs from a 7-day-old fungal strain on YMA medium (2 g/liter yeast extract, 10 g/liter malt extract, 18 g/liter agar) were inoculated into 100 ml of TG medium (1% tryptone [Oxoid, Basingstoke, UK], 1% glucose) and cultured at 30°C at 180 rpm for 36 h. The mycelia were harvested and resuspended in 20 ml of a filter-sterilized enzyme solution that contained 120 mg of lysing enzymes (Sigma-Aldrich, St. Louis, MO), 0.4 ml of cellulase (Sigma-Aldrich, St. Louis, MO), and 100 mg of snailase (Solarbio, Beijing, China) in 0.6 M MgSO_4_ at pH 6.0. The suspension was incubated for 4 h at 28°C on a rotary shaker at 180 rpm. Protoplasts were collected by filtering through six layers of sterile lens-cleaning tissue and centrifuged at 1,000 × *g*. The protoplasts were washed twice with KTC (1.2 M KCl, 10 mM Tris-HCl, 50 mM CaCl_2_) solution and, finally, resuspended in the same solution.

The protoplast-based protocol for the disruption of the targeted genes in A. oligospora was performed as described previously (16). About 150 μl protoplasts (circa 8.0 × 10^7^/ml) was mixed with 10 μg linear DNA in a 1.5-ml centrifuge tube. After 30 min of incubation on ice, 600 μl of PTC (50 mM CaCl_2_, 20 mM Tris-HCl, 50% polyethylene glycol 6000, pH 7.5) was added to the mixture and the components were mixed gently. After incubation at 28°C for 1 h and regeneration for 12 h, the putatively transformed protoplasts were plated onto PDAS medium (PDA supplemented with 5 g/liter molasses, 0.6 M saccharose, 0.3 g/liter yeast extract, 0.3 g/liter tryptone, and 0.3 g/liter casein peptone) containing 200 μg/ml of hygromycin B. Transformed colonies were selected after incubation at 28°C for 6 to 8 days, and every single colony was transferred to a new plate containing TYGA medium (10 g/liter tryptone, 10 g/liter glucose, 5 g/liter yeast extract, 5 g/liter molasses, 18 g/liter agar) with 200 μg/ml of hygromycin B. After incubation for 5 days at 28°C, the genomic DNA of the putative transformants was extracted and was verified by PCR to check for the integration of the genes in the genome. Five mutants deficient in these PKS genes were screened out and confirmed by PCR. Knockout of the PKS I-2 gene (*AOL_s00079g496*) was further confirmed by Southern blot analysis. Southern analysis was carried out according to the instructions provided by the chemiluminescent nucleic acid detection module (Thermo, Rockford, IL, USA). The primer pair KS-5f (TGTATTCCGTTTCGGTCTGC) and KS-3r (TTGAACCAACACGATTCTGC) was used as the Southern hybridization probe, and restriction enzyme AgeI was used to digest the genomic DNA of the wild-type A. oligospora strain and the Δ*AOL_s00079g496* mutant for Southern analysis. All the mutants were maintained and cultured on the same media in the same way as the wild-type strain. The metabolites from cultures of these mutants and the wild-type strain were extracted and analyzed by HPLC and GC-MS methods. GC-MS analysis was performed as described above in “Metabolomic profiling.”

### HPLC analysis.

HPLC analysis was carried out using an HP 1200 unit (Agilent, Waldbronn, Germany) with a Capcell Pak C_18_ column (4.6 by 250 mm; particle size, 5 μm; Shiseido, Tokyo, Japan). Mobile phase A was 0.1% formic acid in water, and mobile phase B was 0.1% formic acid in acetonitrile. The LC conditions were described previously (29) and were manually optimized on the basis of separation patterns as the following gradient program of mobile phase B: 0 min, 10% mobile phase B; 2 min, 10% mobile phase B; 10 min, 25% mobile phase B; 30 min, 35% mobile phase B; 35 min, 50% mobile phase B; 45 min, 90% mobile phase B; 47 min, 10% mobile phase B; and 49 min, 10% mobile phase B. UV spectra were recorded at 220 to 400 nm.

## Supplementary Material

Supplemental material
