# Supplementary material for: Integrated Metabolomics and Morphogenesis Reveal Volatile Signaling of the Nematode-Trapping Fungus Arthrobotrys oligospora
Source: Appl Environ Microbiol. 2018 Apr 16;84(9):e02749-17. doi: 10.1128/AEM.02749-17 (PMC5930339; doi:10.1128/AEM.02749-17)
Supplement: Supplemental material [file supp_84_9_e02749-17__index.html]

Supplemental material 

# Integrated Metabolomics and Morphogenesis Reveal Volatile Signaling of the Nematode-Trapping Fungus Arthrobotrys oligospora

## Supplemental material

- Supplemental file 1 -

  GC-MS profiles of the methanol extracts of *A. oligospora* YMF1.01883 (Fig. S1); GC-MS profiles (Tables S1, S2, and S7); compounds detected by GC-MS analysis of methanol extracts of *A. oligospora* YMF1.01883 (Tables S3 and S4); lists of varied metabolites and their abundance with the time course from the saprophytic to the predacious lifestyle (Tables S5 and S6); HPLC and GC-MS profiles of the five mutants of PKS genes and *A. oligospora* wild type (Table S8); PCR results for transformants (Fig. S2); Southern analysis (Fig. S3).

  PDF, 4.7M
