# Supplementary material for: Integrated Metabolomics and Morphogenesis Reveal Volatile Signaling of the Nematode-Trapping Fungus Arthrobotrys oligospora
Source: Appl Environ Microbiol. 2018 Apr 16;84(9):e02749-17. doi: 10.1128/AEM.02749-17 (PMC5930339; doi:10.1128/AEM.02749-17)
Supplement: Supplemental material [file AEM.02749-17_zam009188466s1.pdf]

Supporting Information

Integrated Metabolomics and Morphogenesis Reveals Volatile Signaling of the Nematode-Trapping Fungus *Arthrobotrys oligospora*

Bai-Le Wang,[1] Yong-Hong Chen,[1] Jia-Ning He,[1] Hua-Xi Xue,[1] Ni Yan,[1] Zhi-Jun Zeng,[1] Joan W. Bennett,[2] Ke-Qin Zhang,[1,]* Xue-Mei Niu[1,]*

[1]State Key Laboratory for Conservation and Utilization of Bio-Resources, Yunnan University, Kunming, 650091, People's Republic of China

[2]Department of Plant Biology and Pathology, Rutgers University, New Jersey 08901, United States of America

*Corresponding author (Tel: 86-871-65032538; Fax: 86-871-65034838: E-mail: xmniu@ynu.edu.cn or kqzhang@ynu.edu.cn)

Fig. S1. GC-MS profiles of the methanol extracts of *A. oligospora* YMF1.01883 on CMA (up) and on PDA (down) under direct and non-direct contact with nematodes at 24h.

Table S1.GC-MS profiles of the methanol extracts of *A. oligospora* YMF1.01883 on PDA under direct and non-direct contact with nematodes during 6-144h. Black: Non-Direct cohabiting with live nematode (NDC-L), Purple: Non-Direct cohabiting with dead nematode (NDC-D), Green: Direct cohabiting with nematode (DC), Red: Control (CON)

Table S2.GC-MS profiles of the methanol extracts of *A. oligospora* YMF1.01883 on CMA under direct and non-direct contact with nematodes during 6-144h. Black: Non-Direct cohabiting with live nematode (NDC-L), Purple: Non-Direct cohabiting with dead nematode (NDC-D), Green: Direct cohabiting with nematode (DC), Red: Control (CON)

Table S3. The compounds detected by GC-MS analysis of methanol extracts of *A. oligospora* YMF1.01883 on PDA under direct and non-direct contact with nematodes during 6-144h.

Table S4. The compounds detected by GC-MS analysis of methanol extracts of *A. oligospora* YMF1.01883 on CMA under direct and non-direct contact with nematodes during 6-144h.

Table S5. The list of the varied metabolites and their abundance with the time course from the saprophytic to the predacious lifestyle of the fungus grown on PDA.

Table S6. The list of the varied metabolites and their abundance with the time course from the saprophytic to the predacious lifestyle of the fungus grown on CMA.

Table S7. GC-MS profiles of metabolites of nematodes under non-direct contact with *A. oligpspora* YMF1.01883 in CMA during 6-144h

Table S8. HPLC and GC-MS profiles of the five mutants of PKS genes and *A. oligospora* wildtype.

Fig. S2. Five transformants were screened and confirmed by the PCR method. W, wild-type strain; M, marker. (A) lanes 7,10,11,12,17 and 18, six *ΔAOL_s00043g287* mutants. (B) lanes 1 and 2, two Δ*AOL_s00043g828* mutants. (C) lanes 1 and 2, two Δ*AOL_s00215g283* mutants. (D) lanes 1-6, 8, 11, 14-18, 21 and 22, fifteen Δ*AOL_s00079496* mutants. (E) lanes 2,4,5,9-11,12 and 18, eight Δ*AOL_s00215g926* mutants.

Fig. S3. Southern analysis of the wild-type strain (WT) and the Δ*AOL_s00079g496* mutant (M). The genomic DNA was digested using AgeI.

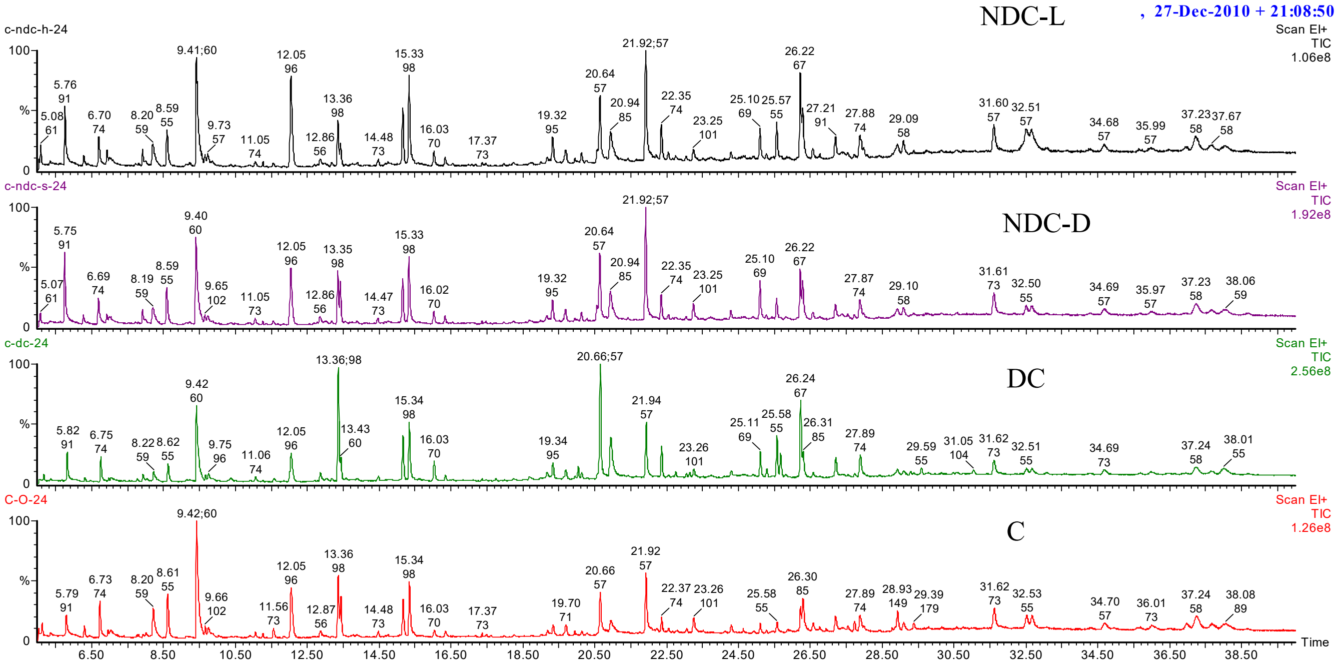

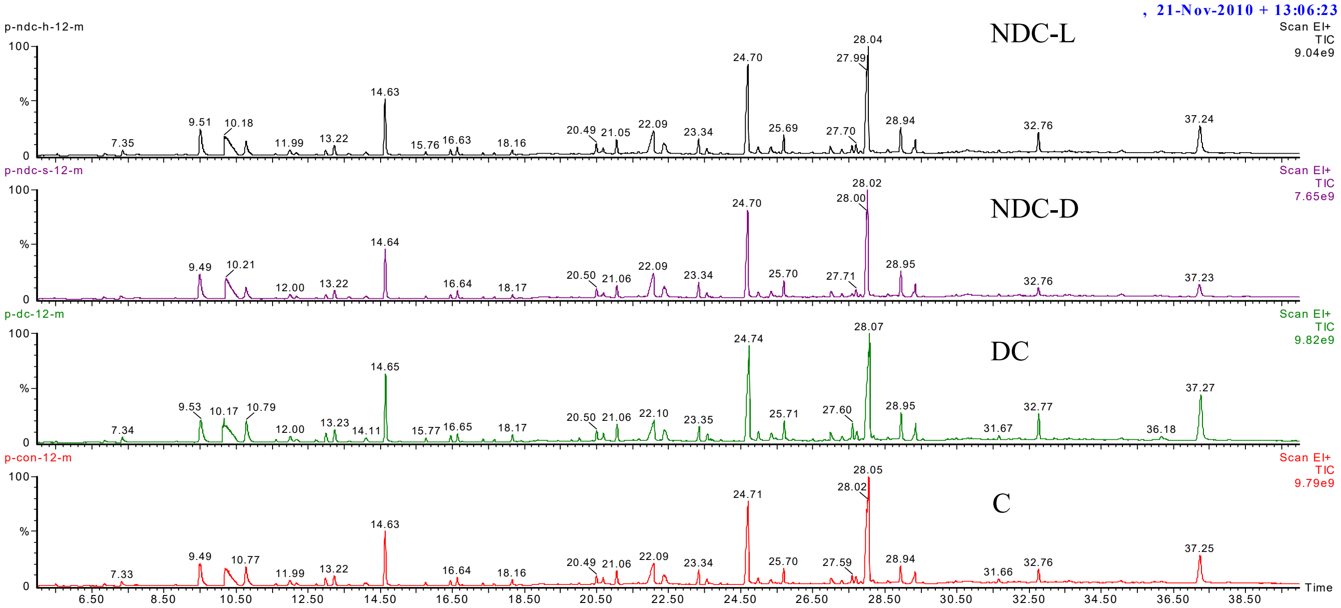

Fig. S1. GC-MS profiles of the methanol extracts of *A. oligospora* YMF1.01883 on CMA (up) and on PDA (down) under direct and non-direct contact with nematodes at 24h. *A. oligospora* growing under non-direct contact live nematodes (NDC-L); *A. oligospora* growing under non-direct contact with dead nematodes (NDC-D); *A. oligospora* growing under direct contact

with nematodes (DC); *A. oligospora* growing without nematodes in 144h as control group (C).

Table S1.GC-MS profiles of the methanol extracts of *A. oligospora* YMF1.01883 on PDA under direct and non-direct contact with nematodes during 6-144h

Black: Non-Direct cohabiting with live nematode (NDC-L), Purple: Non-Direct cohabiting with dead nematode (NDC-D), Green: Direct cohabiting with nematode (DC), Red: Control (CON)

Time	GC-MS profiles
6h	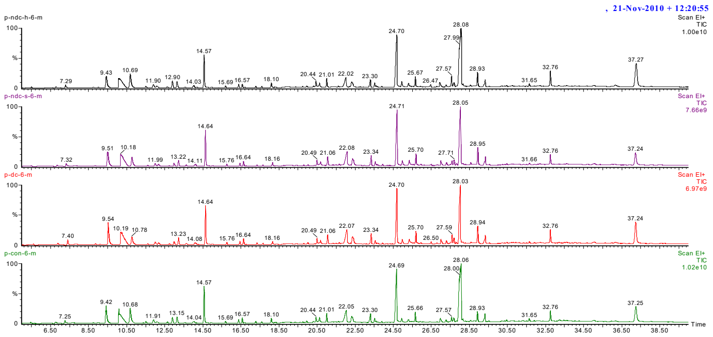
12h	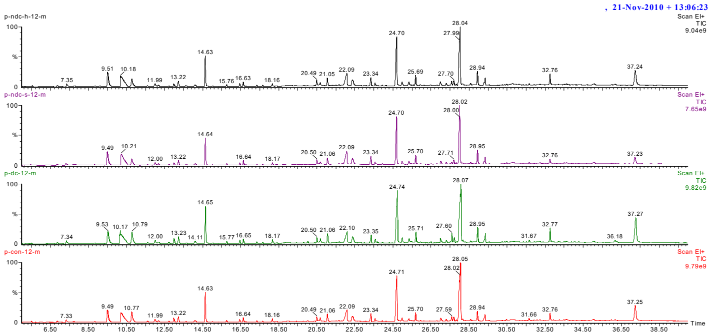
18h	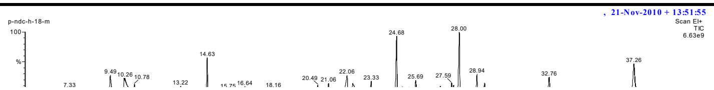

24h

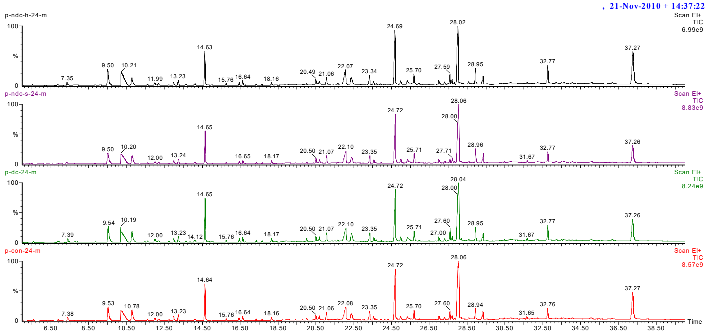

, 21-Nov-2010 + 14:37:22
p-ndc-h-24-m
Scan EI+ TIC 6.99e9
p-ndc-s-24-m
Scan EI+ TIC 8.83e9
p-dc-24-m
Scan EI+ TIC 8.24e9
p-con-24-m
Scan EI+ TIC 8.57e9
Time

30h

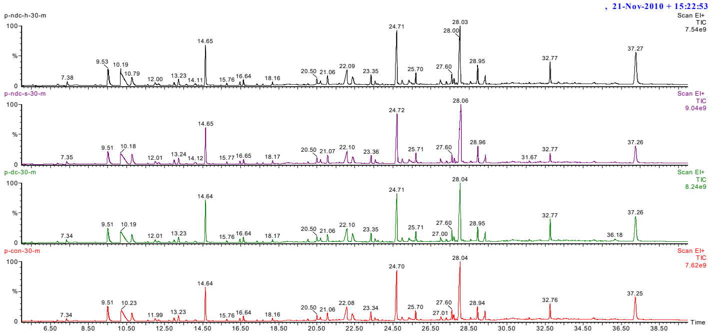

, 21-Nov-2010 + 15:22:53
p-ndc-h-30-m
Scan EI+ TIC 7.54e9
p-ndc-s-30-m
Scan EI+ TIC 9.04e9
p-dc-30-m
Scan EI+ TIC 8.24e9
p-con-30-m
Scan EI+ TIC 7.62e9
Time

36h

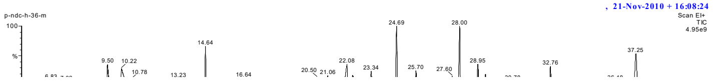

, 21-Nov-2010 + 16:08:24
p-ndc-h-36-m
Scan EI+ TIC 4.95e9

42h

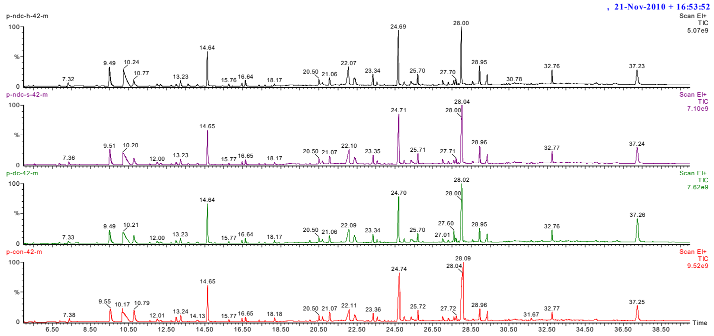
21-Nov-2010 + 16:53:52
p-ndc-h-42-m
p-ndc-s-42-m
p-dc-42-m
p-con-42-m
Time

48h

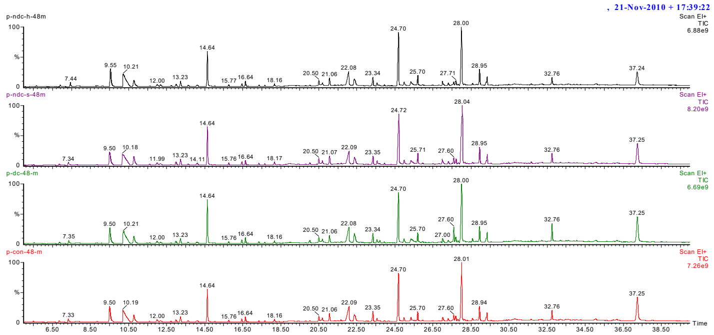
21-Nov-2010 + 17:39:22
p-ndc-h-48m
p-ndc-s-48m
p-dc-48-m
p-con-48-m
Time

72h

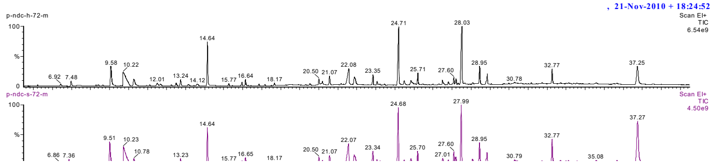
21-Nov-2010 + 18:24:52
p-ndc-h-72-m
p-ndc-s-72-m

96h

120h

144h

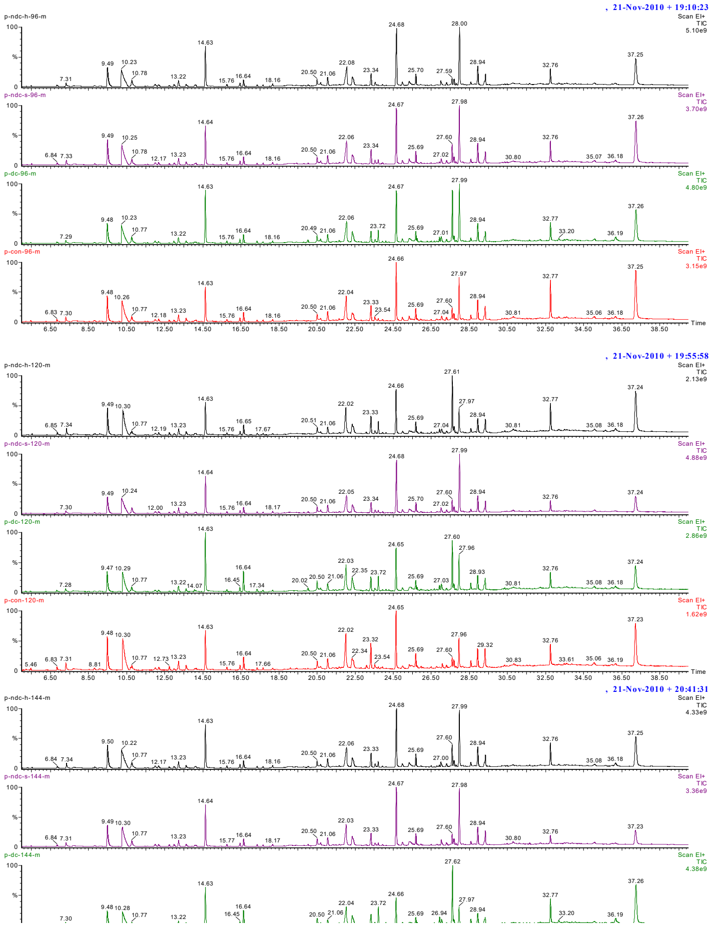

Table S2.GC-MS profiles of the methanol extracts of *A. oligospora* YMF1.01883 on CMA under direct and non-direct contact with nematodes during 6-144h

Black: Non-Direct cohabiting with live nematode (NDC-L), Purple: Non-Direct cohabiting with dead nematode (NDC-D), Green: Direct cohabiting with nematode (DC), Red: Control (CON)

Time	GC-MS profiles
6h	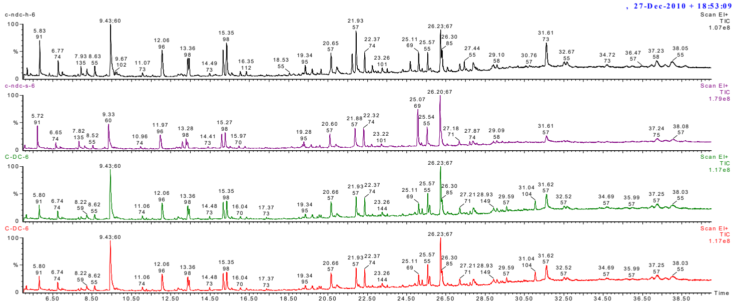
12h	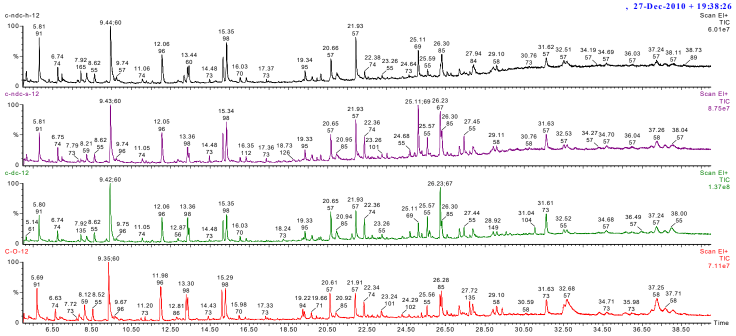
18h	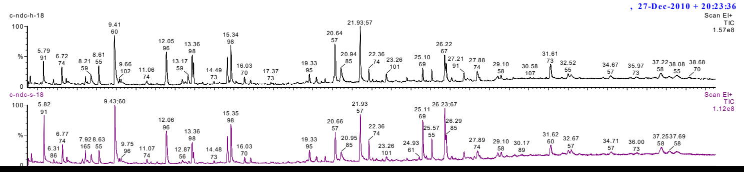

24h

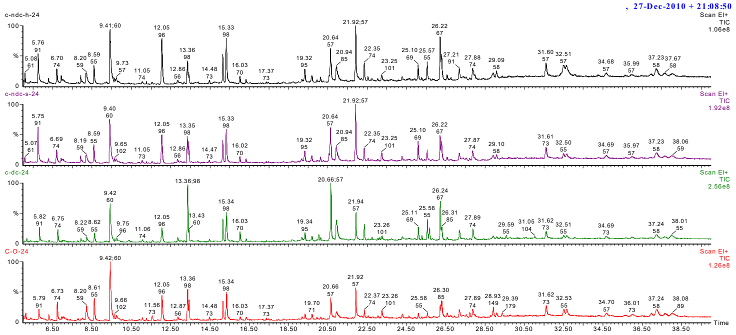
27-Dec-2010 + 21:08:50
c-ndc-h-24
c-ndc-s-24
c-dc-24
C-O-24
Scan EI+
TIC
Time

30h

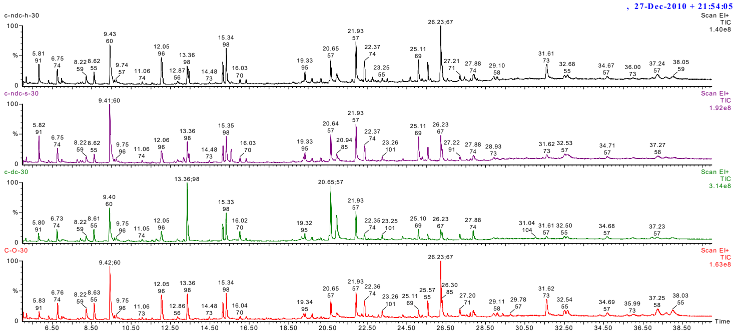
27-Dec-2010 + 21:54:05
c-ndc-h-30
c-ndc-s-30
c-dc-30
C-O-30
Scan EI+
TIC
Time

36h

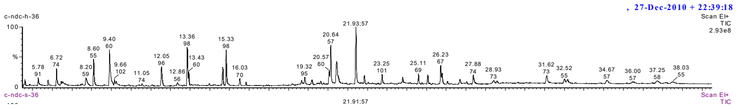
27-Dec-2010 + 22:39:18
c-ndc-h-36
c-ndc-s-36
Scan EI+
TIC

42h

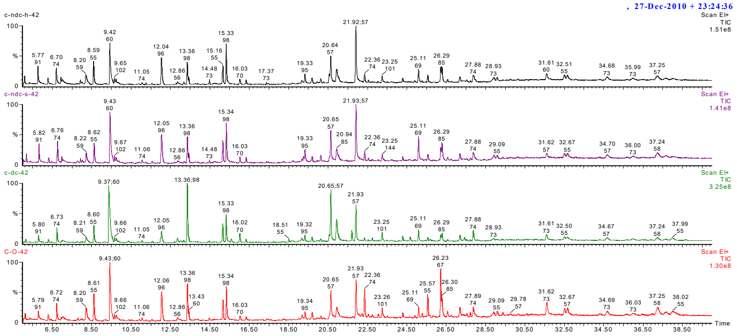
27-Dec-2010 + 23:24:36
c-ndc-h-42
c-ndc-s-42
c-dc-42
C-O-42
Scan EI+
TIC
Time

48h

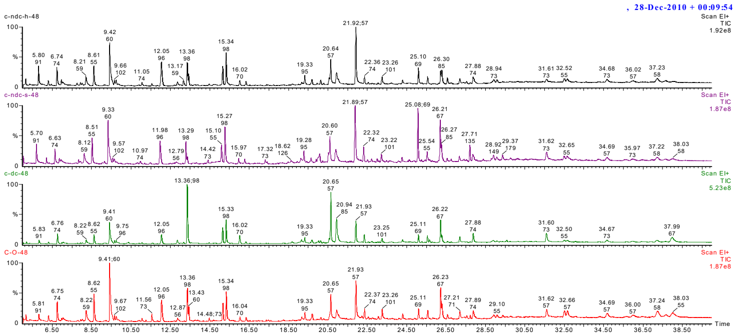
28-Dec-2010 + 00:09:54
c-ndc-h-48
c-ndc-s-48
c-dc-48
C-O-48
Scan EI+
TIC
Time

72h

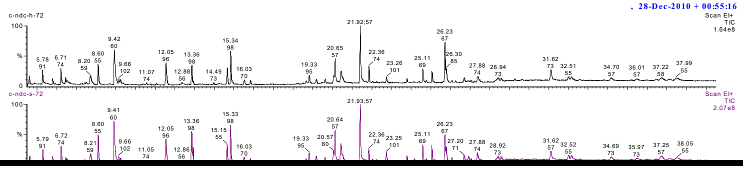
28-Dec-2010 + 00:55:16
c-ndc-h-72
c-ndc-s-72
Scan EI+
TIC

96h

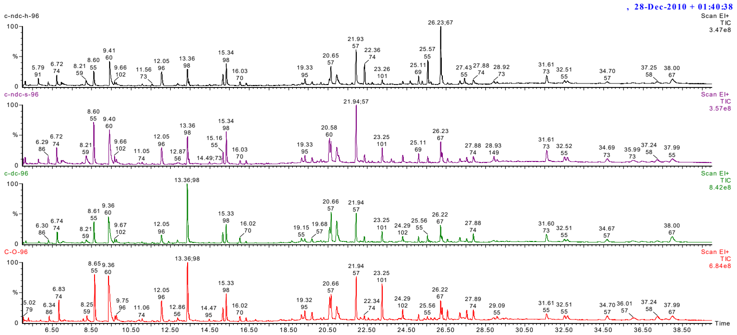

120h

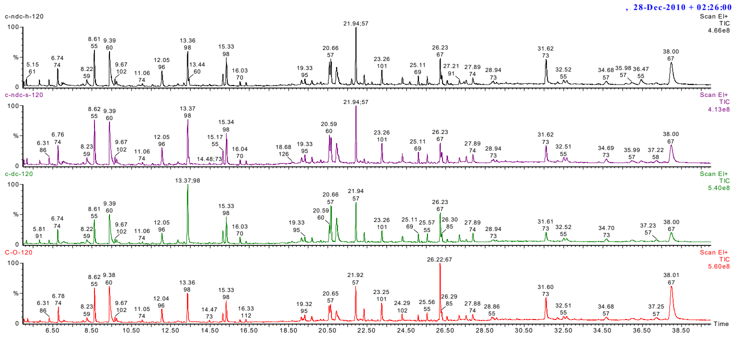

144h

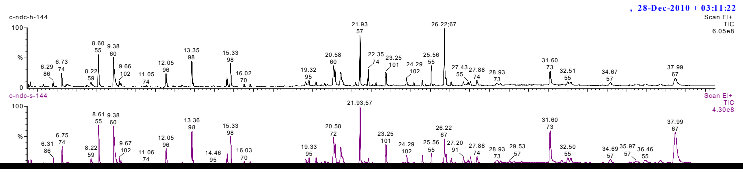

Table S3. The compounds detected by GC-MS analysis of methanol extracts of *A. oligospora* YMF1.01883 on PDA under direct and non-direct contact with nematodes during 6-144h.

No	Rt (min)	Hit Name	Molecular Formula	Structure
1	6.849	Ethyl-1-propenyl ether	$C_5H_{10}O$	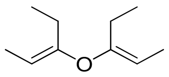
2	7.339	N-nitrosodi methylamine	$C_2H_6N_2O$	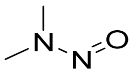
3	7.739	Acetic acid methoxy	$C_3H_6O_3$	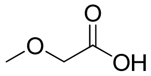
4	8.825	1-Hydroxy-2-butanone	$C_4H_8O_2$	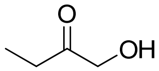
5	9.095	3-Ethoxy-1,2-propane	$C_5H_{12}O_3$	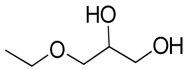
6	9.480	2,2-Bioxirane	$C_4H_6O_2$	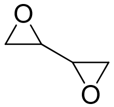
7	10.205	®-Urea	CH_4NO_2	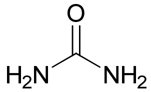
8	10.606	Ethanone, 1-(2-furanyl)-	$C_6H_6O_2$	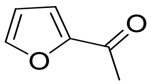
9	10.781	(M)Furfural	$C_5H_4O_2$	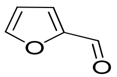

10	11.996	2,4-Dihydroxy-2,5-dimethyl-3(2H)-furan-3-one	$C_6H_8O_4$	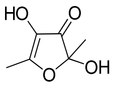
11	12.181	Ethanol,2-nitro-propionate(ester)	$C_5H_9NO_4$	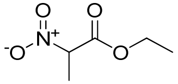
12	12.726	®-Propanoic acid 2-methyl-	$C_4H_8O_2$	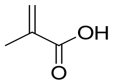
13	12.980	®-2-Furancarboxaldehyde,5-methyl-	$C_6H_6O_2$	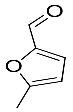
14	13.222	(M)2-cyclopentene-1,4-dione	$C_5H_4O_2$	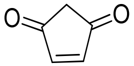
15	13.482	2,3,4,5,6,7-hexahydroxyheptanal	$C_7H_{14}O_7$	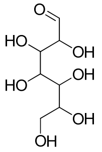
16	13.622	(Z)-3-(2-((3H-indol-3-yl)methylene)hydroaziny)-5-methyl-4H-1,2,4-triazol-4-amin	$C_{12}H_{13}N_7$	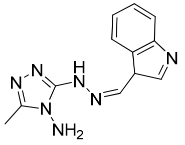
17	14.077	Butyrolactone	$C_4H_6O_2$	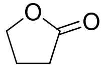
18	14.122	2-propenoic acid	$C_3H_4O_2$	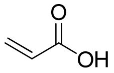
19	14.257	Benzeneethanaminea-methyl	$C_9H_{13}N$	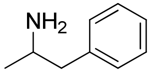

20	15.017	1,2-Cyclopentanediol ,3-methyl-	$C_6H_{12}O_2$	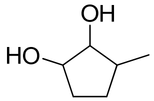
21	15.763	2-Furanmethanol, 5-methyl-	$C_6H_8O_2$	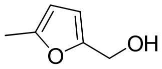
22	16.380	2-Cyclopenten-1-one,2-hydroxy	$C_5H_6O_2$	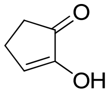
23	16.448	2(5H)-Furanone	$C_4H_4O_2$	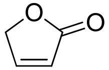
24	16.773	Nona-3,5-dien-2-one	$C_9H_{14}O$	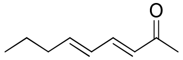
25	16.913	3,3-Dimethyl -2-pentanol	$C_7H_{16}O$	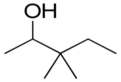
26	17.568	2-Cyclopenten-1-one, 2-hydroxy-3-methyl-	$C_6H_8O_2$	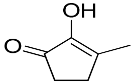
27	18.334	Geranyl acetate	$C_{12}H_{20}O_3$	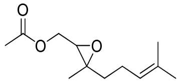
28	18.404	2-(2-aminoacetamido) 3-hydroxybutanoic acid	$C_6H_{12}N_2O_4$	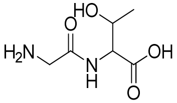
29	18.759	(M)methyl,6 -oxoheptanoate	$C_8H_{14}O_3$	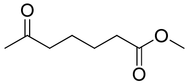

30	18.954	Thiophene,tetrohydro -3-methyl-	$C_5H_{10}S$	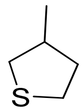
31	19.109	O-acetyl-L-serine	$C_5H_9NO_4$	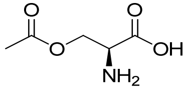
32	19.429	(M)Propanoic acid,3-(acetylthio)-2-methyl	$C_6H_{10}O_3S$	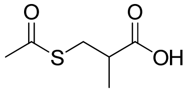
33	19.679	2-hydroxyhexadecyl butanoate	$C_{20}H_{40}O_3$	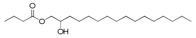
34	19.794	Benzenemethanol, 4-hydroxy-	$C_7H_8O_2$	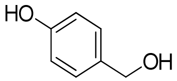
35	19.884	®-6-Dodecanol acetate	$C_{14}H_{28}O_2$	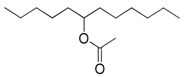
36	20.014	3-Hydroxy-2-methyl-4H-pyran-4-one	$C_6H_6O_3$	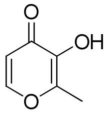
37	20.364	9-Oxobicyclo(3,3,1)n onan-2-ol,acetate	$C_{10}H_{16}O_3$	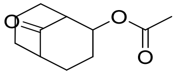
38	20.495	2H-pyran-2,6 -(3H)-dione	$C_5H_4O_3$	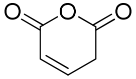
39	20.685	Furyl hydroxymethyl ketone	$C_6H_6O_3$	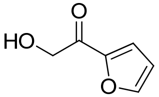
40	21.060	2,5-Dimethyl-4-hydr oxy-3(2H)-furanone	$C_6H_8O_3$	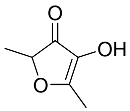

41	21.35	Desulphosinigrin	$C_{10}H_{17}NO_6S$	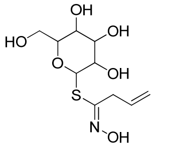
42	21.61	(M)Isosorbide Dinitrate	$C_6O_8N_2O_8$	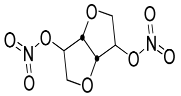
43	22.075	di-Gryceraldehyde dimer	$C_6H_{12}O_6$	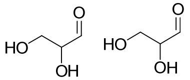
44	22.225	Ally 2-ethyl butyrate	$C_9H_{16}O_2$	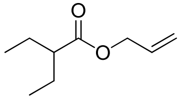
45	22.370	Ethanone, 1-[3-ethyloxiranyl]-	$C_6H_{10}O_2$	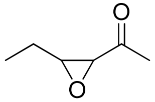
46	22.815	2,4-Dimethyl hexanedioic acid	$C_8H_{14}O_4$	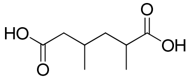
47	22.996	a-D-glucopyranoside, o-a-D-glucopyranosyl -(1,fudarw,3)-β-D-fru ctofuranosyl	$C_{18}H_{32}O_{16}$	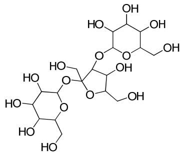
48	23.336	Formic acid 2-propenyl-ester	$C_4H_6O_2$	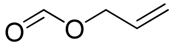
49	23.651	6-Acetyl-β-dimannose	$C_8H_{14}O_7$	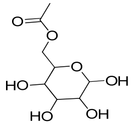

50	23.716	Methyl, 6-oxoheptanoate	$C_8H_{14}O_3$	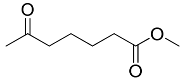
51	23.941	2H-pyran-3(4H)-one, dihydro-6-methyl	$C_6H_{10}O_2$	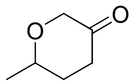
52	24.685	4H-pypron-4-one, 2,3-dihydro,3,5-dihydroxy-6-methy	$C_6H_8O_4$	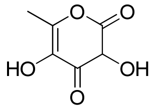
53	25.336	D(+)-Talose	$C_6H_{12}O_6$	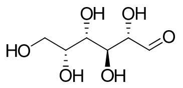
54	25.692	Ethanamine,N-ethyl-N-nitroso-	$C_4H_{10}N_2O$	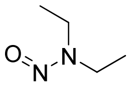
55	25.952	1-Dodecanol,3,7,11-trimethyl-	$C_{15}H_{32}O$	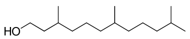
56	26.812	Dodecanoic acid 3-hydroxy-	$C_{12}H_{24}O_3$	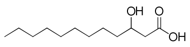
57	26.932	6-Octadecenoic acid methyl ester	$C_{19}H_{36}O_2$	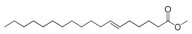
58	27.017	3-Furancarboxylic acid	$C_5H_4O_3$	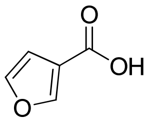
59	27.297	Piperidine-1-dithiocarboxylic acid 2-oxocyclopentyl ester	$C_{11}H_{17}NOS_2$	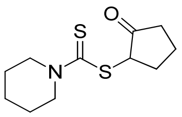
60	27.678	2(3H)-Furanone, 5-heptyldihydro-	$C_{11}H_{20}O_2$	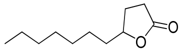

61	27.702	5-Hydroxymethyl dihydrofuran-2-one	$C_5H_8O_3$	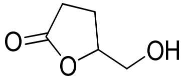
62	27.967	2-Furancarboxaldehy de,5-(hydroxymethyl)	$C_6H_6O_3$	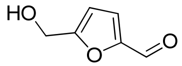
63	28.022	(R)-1-phenyl-1,2-etha nediol	$C_8H_{10}O_2$	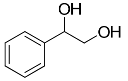
64	28.183	1-Dodecanol, 3,7,11-trimethyl-	$C_{15}H_{32}O$	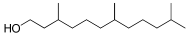
65	28.588	Benzeneacetic acid 4-tetradecyl ester	$C_{22}H_{36}O_2$	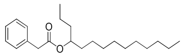
66	28.938	2-Pentenoic acid, 3-methyl,methyl ester	$C_7H_{12}O_2$	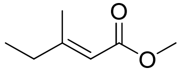
67	29.333	DL-proline 5-oxo, methyl ester	$C_6H_9NO_3$	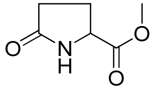
68	29.555	Z-(13,14-Epoxy) tetradec-11-en-1-ol acetate	$C_{16}H_{28}O_3$	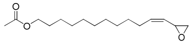
69	30.473	Pyrrolizin-1,7-dione-6-carboxyli acid methyl ester	$C_9H_{11}NO_4$	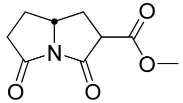
70	31.124	Dodecanoic acid 3-hydroxy	$C_{12}H_{24}O_3$	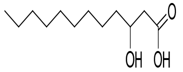
71	31.659	Uric acid	$C_5H_4N_4O_3$	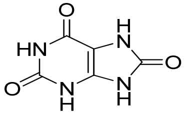
72	32.224	1,3-Dioxolane, 4-ethyl-4-methyl-	$C_{21}H_{42}O_2$	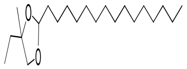

		2-pertadecyl		
73	32.779	n-Hexadecanoic acid	$C_{16}H_{32}O_2$	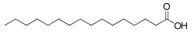
74	33.225	9.10-Secocholesta-5,7-10(19)-triene-1,3-diol 25-[(trimethylsilyl)oxcy]	$C_{27}H_{44}O_3$	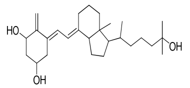
75	33.485	1-Dodecanoc, 3,7,11-trimethyl	$C_{15}H_{32}O$	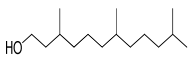
76	34.065	[1,1-bicyclopropyl]-2-octanoic acid 2-hexyl-methyl ester	$C_{21}H_{38}O_2$	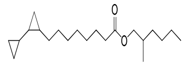
77	34.70	Dodecanoic acid 3-hydroxy	$C_{12}H_{24}O_3$	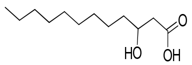
78	35.05	2-Myristynoyl pantetheine	$C_{25}H_{44}N_2O_5$	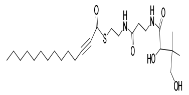
79	36.661	2-Hexadecanol	$C_{16}H_{34}O$	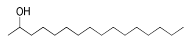
80	36.661	1,3-methyl-7,8,9,11,12,13,14,15,16,17-decahydro-6H-cyclopenta[a]phenanthren-17-	$C_{18}H_{24}O$	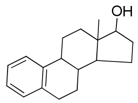

Table S4. The compounds detected by GC-MS analysis of methanol extracts of *A. oligospora* YMF1.01883 on CMA under direct and non-direct contact with nematodes during 6-144h.

No	RT (min)	Hit Name	Molecular Formula	Structure

1	5.078	Trifluoroguanidine	$CH_2F_3N_3$	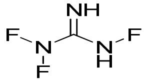
2	5.128	Methane,nitro-	CH_3NO_2	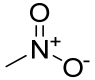
3	5.168	Urea	CH_4N_2O	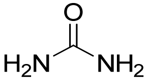
4	5.364	3-cyclopentene-1,2-diol	$C_5H_8O_2$	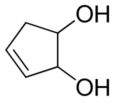
5	6.299	N-(2,4,4-trimethylpentan -2-yl)formamide	$C_9H_{19}NO$	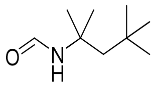
6	6.699	Methyl hex-5-enoate	$C_7H_{12}O_2$	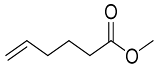
7	6.754	Methyl-(3R)-(-)-5-oxo-3-propylpentanoate	$C_9H_{16}O_3$	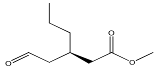
8	6.929	3-Ethoxy-1, 2-propanediol	$C_5H_{12}O_3$	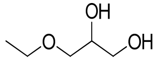
9	7.014	(2R,3R,4R,5R)-2-methoxytetrahydro-2H-pyran-3,4,5-triol	$C_6H_{12}O_5$	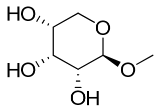
10	7.915	6-methoxy-9H-purin-2-amine	$C_6N_7N_5O$	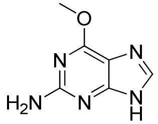
11	8.220	Acetic acid,hydroxyl methyl ester	$C_3H_6O_3$	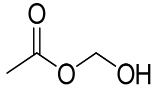

12	8.625	1-Butanol,2-nitro	$C_4H_9NO_3$	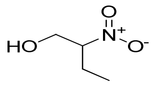
13	9.415	Formic acid methyl ester	$C_2H_4O_2$	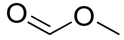
14	9.660	5,10-dithiadispiro [3,1,3,1]decane-5,5,10,10-tetraoxide	$C_8H_{12}O_4S_2$	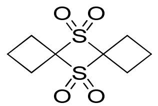
15	9.735	2-furanmethanediol, dipropionate	$C_{11}H_{14}O_5$	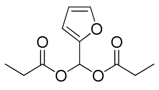
16	9.750	2-Furancarboxaldehyde	$C_5H_4O_2$	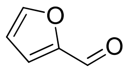
17	10.376	Acetic acid 2-(2-pyrrolidinyli deneamino)-	$C_6H_{10}N_2O_2$	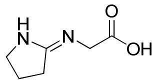
18	11.051	Propanoic acid	$C_3H_6O_2$	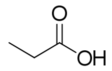
19	11.546	Butanoic acid,2-ethyl	$C_6H_{12}O_2$	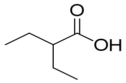
20	11.841	1-methoxycyclohexa-1,3-diene	$C_7H_{10}O$	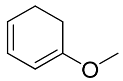
21	12.056	Cyclopent-2-en-1,4-dione	$C_5H_4O_2$	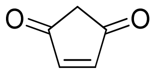
22	12.097	3-hydroxypropano hydrazide	$C_3H_7DN_2O_2$	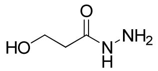
23	12.442	Pentanoic acid	$C_5H_{10}O_2$	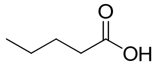
24	12.857	Butanoicacid, 4-hydroxy-	$C_4H_8O_3$	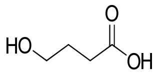

25	12.857	Dihydrofuran-2(3H)-one	$C_4H_6O_2$	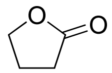
26	13.177	2-propanol,1-(isooctyloxy)2-methyl-	$C_{12}H_{26}O_2$	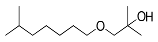
27	13.352	Furan-2-ylmethanol	$C_5H_6O_2$	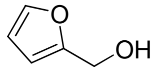
28	13.887	2-Ethylhex-4-en-1-ol	$C_8H_{16}O$	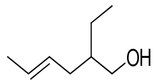
29	14.742	Acetic acid,2,2-[oxybis (2,1-ethanediyloxy)]bis	$C_8H_{14}O_7$	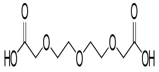
30	15.158	2(5H)-furanone	$C_4H_4O_2$	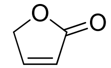
31	15.328	2-Cyclopenten-1-one, 2-hydroxy-	$C_5H_6O_2$	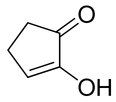
32	16.032	4-cyclopentene-1,2,3-triol,(1a,2a,3a)	$C_5H_8O_3$	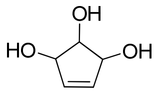
33	16.338	2-cyclopenten-1-one, 2-hydroxy-3-methyl-	$C_6H_8O_2$	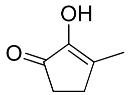
34	16.543	d-mannose	$C_6H_{12}O_6$	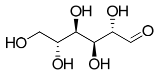
35	17.068	3,6,9,12-Tetraoxadolosan-1-ol	$C_{18}H_{38}O_5$	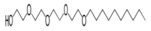
36	17.754	3-Buten-2-one, 4-(3-hydroxy-6,6-dimethyl-2-methylenecyclohexyl)-	$C_{13}H_{20}O_2$	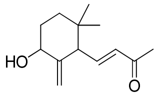

37	18.694	1-4(hydroxyl-5(hydroxymethyl)tetrahydrofuran-2-yl)-5-methylpyrimidine-2,4-(1H,3H)-dione	$C_{10}H_{14}NO_5$	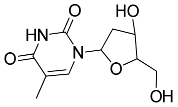
38	19.189	2-(3-hydroxy-propyl)-cyclohexane-1,3-dione	$C_9H_{14}O_3$	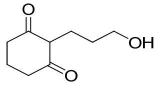
39	19.324	2-furancarbonsaeuro, methyl ester	$C_6H_6O_3$	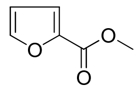
40	19.649	n-butyric acid 2-ethylhexyl ester	$C_{12}H_{24}O_2$	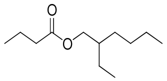
41	20.040	Octadecanoic acid methyl ester	$C_{19}H_{38}O_2$	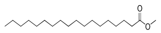
42	20.145	Nonan-1-ol	$C_9H_{20}O$	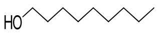
43	20.290	2,4:3,5-Dimethylene-1-iditol	$C_8H_{14}O_6$	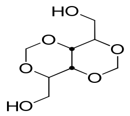
44	20.370	Hexahydrofuro[3,2,6]furan-3,6-diyldinitrate	$C_8H_{14}O_6$	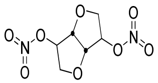
45	20.655	Axirane,[(2-propenylox)methyl]-	$C_6H_{10}O_2$	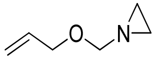
46	20.950	Ethanone, 1-(3-ethyloxianyl)-	$C_6H_{10}O_2$	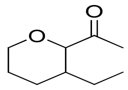
47	21.925	Formic acid, 2-propenyl ester	$C_4H_6O_2$	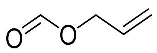

48	22.366	Methyl palmitate	$C_{17}H_{34}O_2$	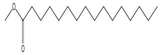
49	22.546	3-thiepanol	$C_6H_{12}OS$	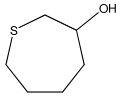
50	22.761	9-Hexadecenoic acid methyl ester	$C_{17}H_{32}O_2$	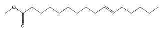
51	23.251	2,3-dihydro-3,5-dihydroxy-6-methyl-4H-pyran-4-one	$C_6H_8O_4$	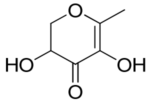
52	23.521	3,6,9,12-Tetraoxadolosan-1-ol	$C_{18}H_{38}O_5$	
53	23.751	a-D-glucopyranoside,o-a-D-glucopyranosyl-(1,fudarw,3)-β-D-fructofuranosyl	$C_{18}H_{32}O_{16}$	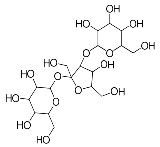
54	25.112	1,4;3,6-Dianhydro-a-d-glucopyranose	$C_6H_8O_4$	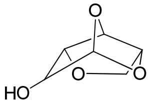
55	25.297	Eicosanoic acid methyl ester	$C_{21}H_{42}O_2$	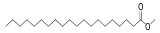
56	25.577	9-Octadecenoic acid (Z),methyl ester	$C_{19}H_{36}O_2$	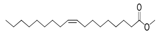
57	26.237	9,12-Octadecadienoic acid (Z,Z)-methyl ester	$C_{19}H_{34}O_2$	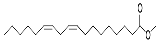
58	27.207	Benzeneacetic acid, decyl ester	$C_{18}H_{28}O_2$	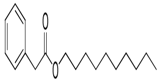

59	27.548	(5,5-Dimethylheptane-1,3-diy)dicyclohexane	$C_{21}H_{40}$	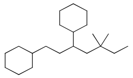
60	27.733	Phenol,4-(1,1,3,3-tetramethylbutyl)-	$C_{14}H_{22}O$	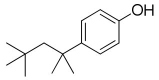
61	27.883	2(3H)-furanone, dihydro-4-hydroxy-	$C_4H_6O_3$	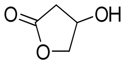
62	28.938	Diisobutyl phthalate	$C_{16}H_{22}O_4$	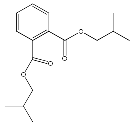
63	29.599	5,8,11,14-Eicosatetraenoic acid methyl ester	$C_{21}H_{34}O_2$	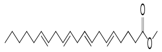
64	31.634	Hexadecanoic acid	$C_{16}H_{32}O_2$	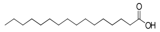
65	32.540	1-pentanol,5-[(Tetrahydro-2H-pyran-2-yl)oxyl]	$C_{10}H_{20}O_3$	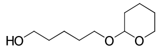
66	32.690	Octaethylene glycol monpdodecyl ether	$C_{28}H_{58}O_9$	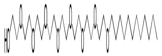
67	34.691	15-Crown-5	$C_{10}H_{20}O_5$	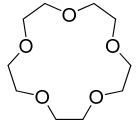
68	37.241	2-[2-(Butoxyethoxy) ethoxy]ethanol	$C_{10}H_{22}O_4$	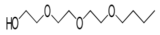
69	37.257	2-Propanone, 1,1-diethoxy-	$C_7H_{14}O_3$	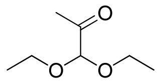
70	37.986	9,12-Octadecadienoic acid(z,z)-	$C_{18}H_{32}O_2$	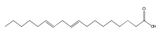

Table S5. The list of the varied metabolites and their abundance with the time course from the saprophytic to the predacious lifestyle of the fungus grown on PDA.

	Hit Name	Molecular Formula	Structure	
1	N-nitrosodimethylamine	$C_2H_6N_2O$	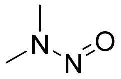	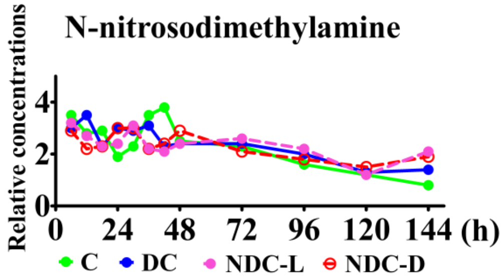
2	1-Hydroxy-2-butanone	$C_4H_8O_2$	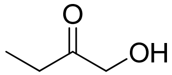	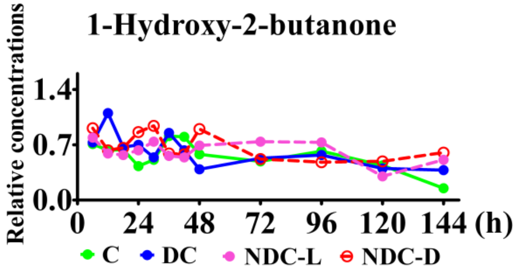

3	1-[3-Ethyloxiranyl]-ethanone	$C_6H_{10}O_2$	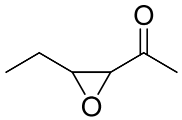	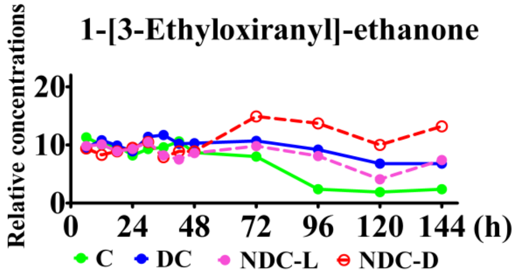
4	3-Ethoxypropane-1,2-diol	$C_5H_{12}O_3$	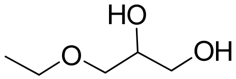	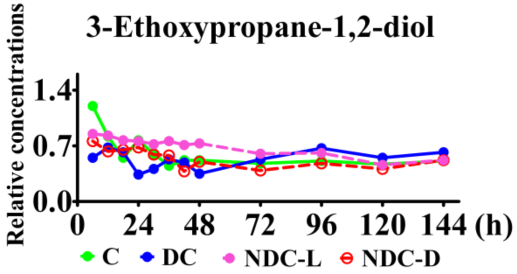
5	Furan-2-carbaldehyde	$C_5H_4O_2$	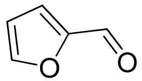	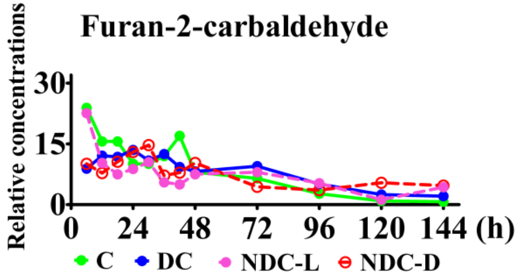

6	5-Methylfuran-2-carbaldehyde	$C_6H_6O_2$	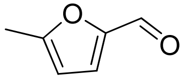	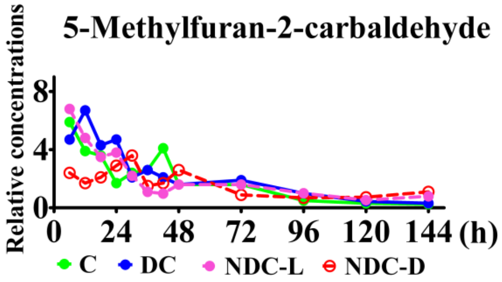
7	2,4-Dihydroxy-2,5-dimethyl-3(2*H*)-furan-3-one	$C_6H_8O_4$	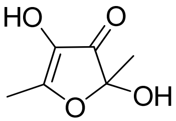	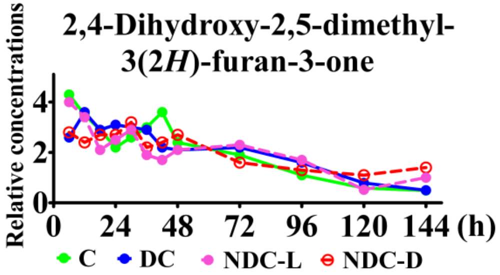
8	3-Hydroxy-2-methyl-4*H*-pyran-4-one	$C_6H_6O_3$	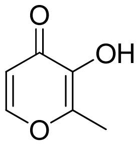	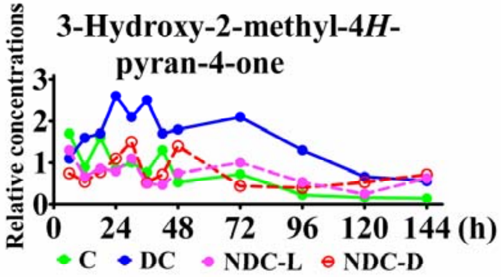

9	2*H*-pyran-2,6(3*H*)-dione	$C_5H_4O_3$		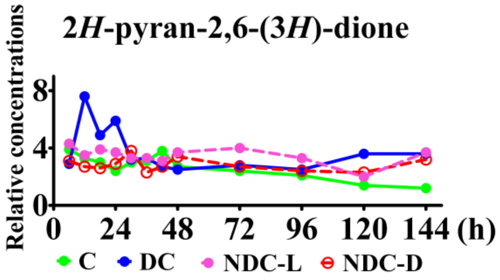
10	6-Acetyl-*β*-dimannose	$C_8H_{14}O_7$	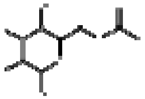	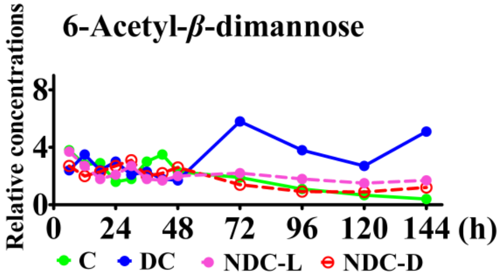
11	D-(+)-Talose	$C_6H_{12}O_6$		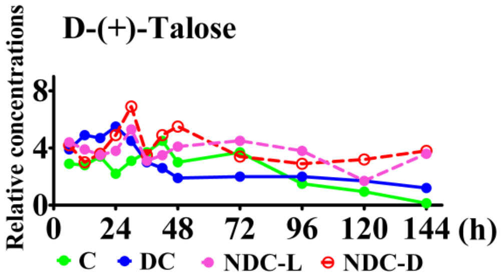

12	(9Z,12Z)-Methyl octadeca-9,12-dienoate	$C_{19}H_{34}O_2$	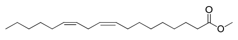	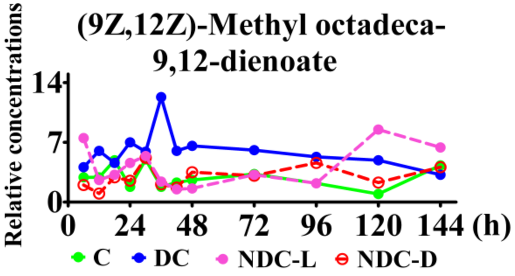
13	17-Octadecynoic acid	$C_{18}H_{32}O_2$	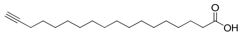	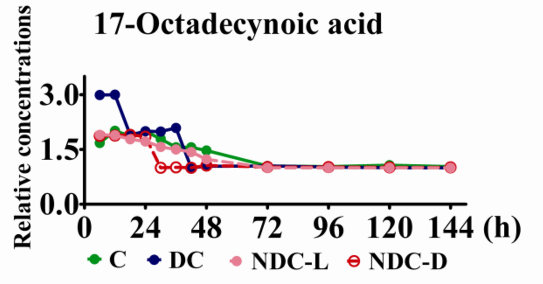
14	n-Hexadecanoic acid	$C_{16}H_{32}O_2$	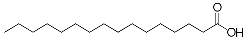	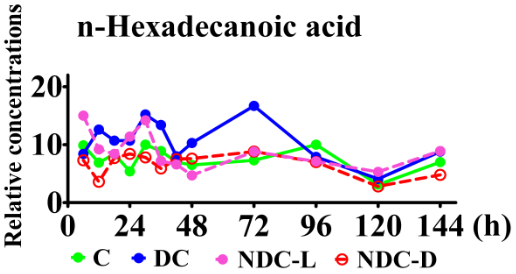

15	(R)-1-phenyl-1,2-ethanediol	$C_8H_{10}O_2$	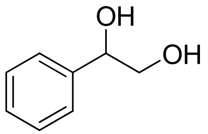	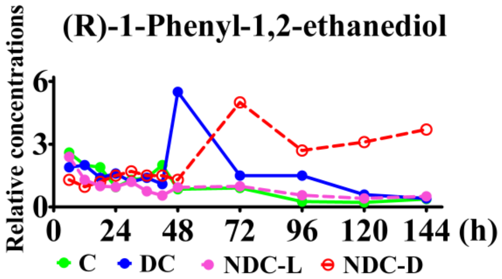
16	Uric acid	$C_5H_4N_4O_3$	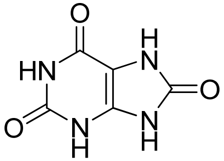	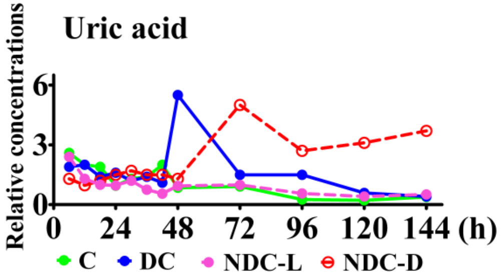

Table S6. The list of the varied metabolites and their abundance with the time course from the saprophytic to the predacious lifestyle of the fungus grown on CMA.

	Hit Name	Molecular Formula	Structure	
1	Nitromethane	CH_3NO_2	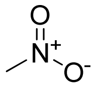	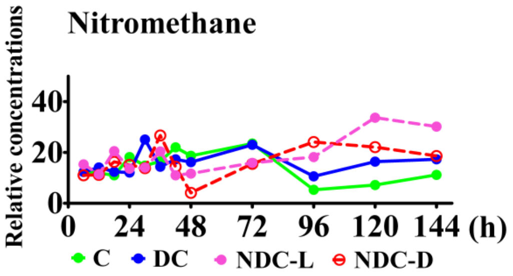
2	Methyl formate	$C_2H_4O_2$	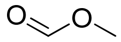	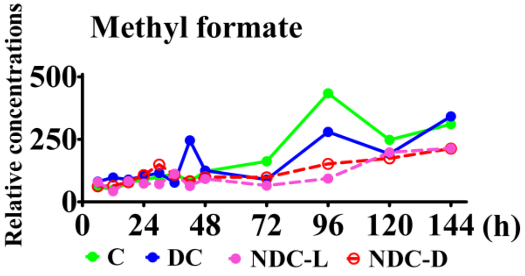
3	2-Propenyl formate	$C_4H_6O_2$	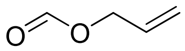	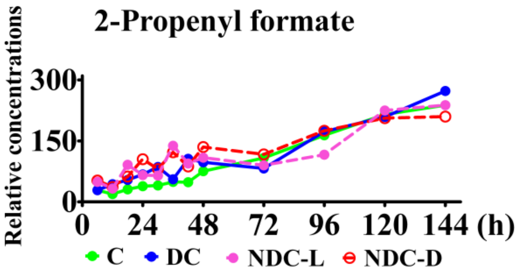

4	2-Pentanone	$C_5H_{10}O$	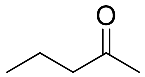	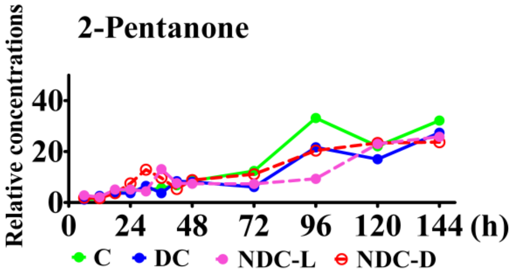
5	Propanoic acid	$C_3H_6O_2$	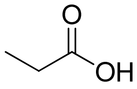	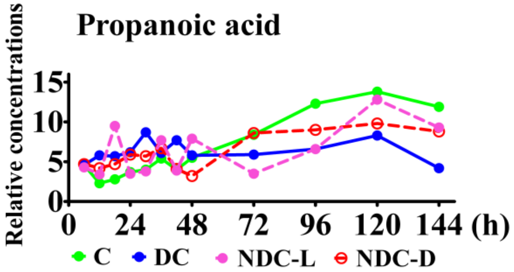
6	Methyl 2-hydroxyacetate	$C_3H_6O_3$	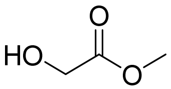	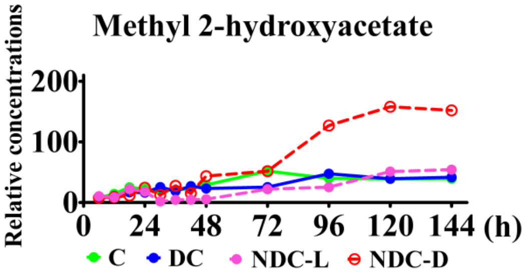

7	4-Hydroxybuta noic acid	$C_4H_8O_3$	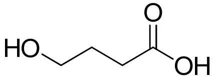	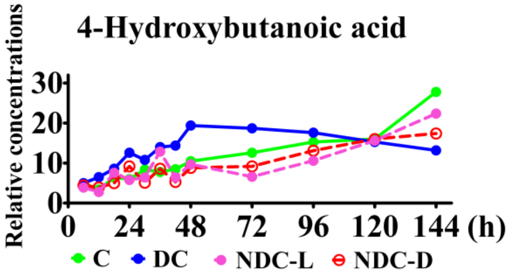
8	3-Ethoxy-1,2-p ropanediol	$C_5H_{12}O_3$	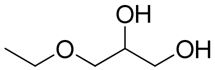	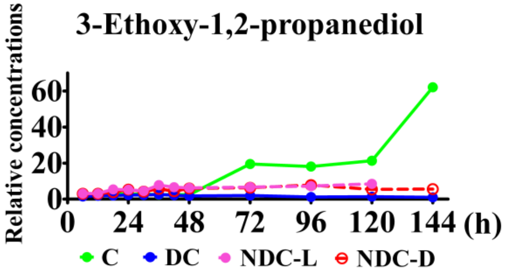
9	1,1-Diethoxy-2 -propanone	$C_7H_{14}O_3$	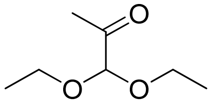	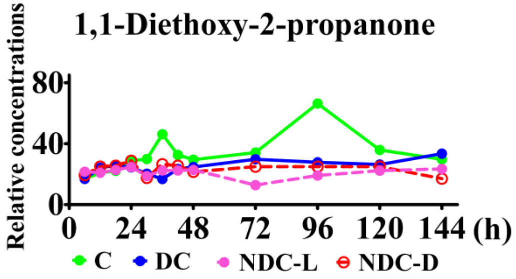

10	Ethane-1,2-diyldiacrylate	$C_8H_{10}O_4$		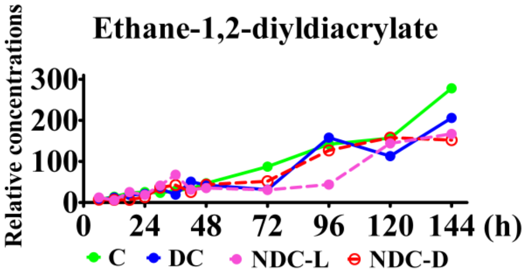
11	1-(Allyloxymethyl)aziridine	$C_6H_{10}O_2$	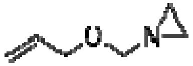	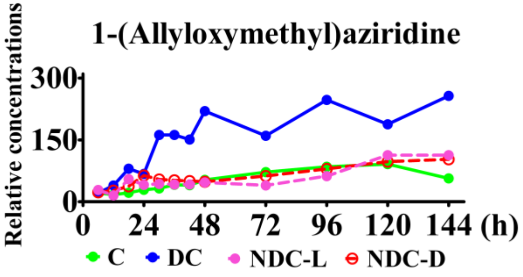
12	2(5*H*)-furanone	$C_4H_4O_2$		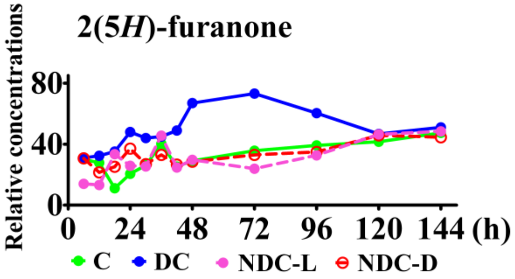

13	2-Hydroxy-2-cyclopenten-1-one	$C_5H_6O_2$	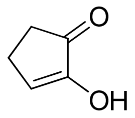	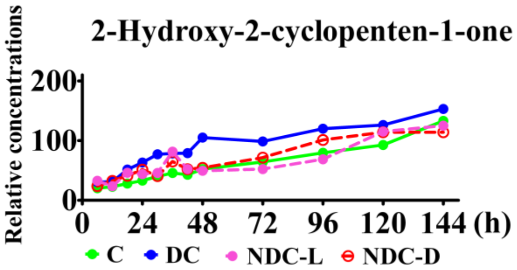
14	Furan-2-carbaldehyde	$C_5H_4O_2$	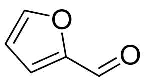	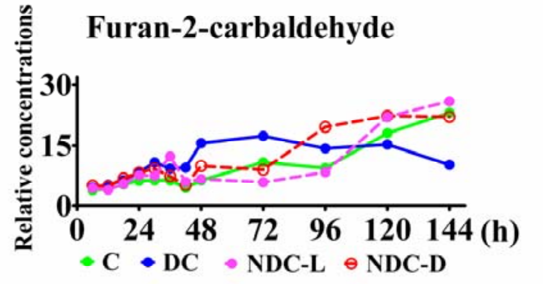
15	Furan-2-ylmethanol	$C_5H_6O_2$	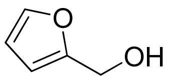	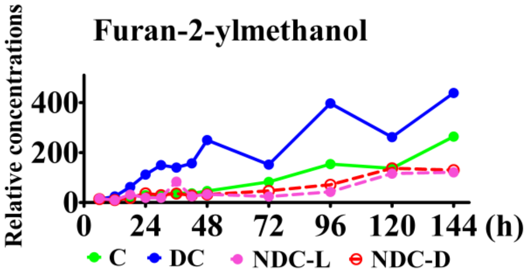

16	Methyl furan-2-carboxylate	$C_6H_6O_3$	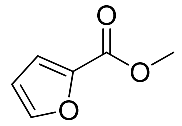	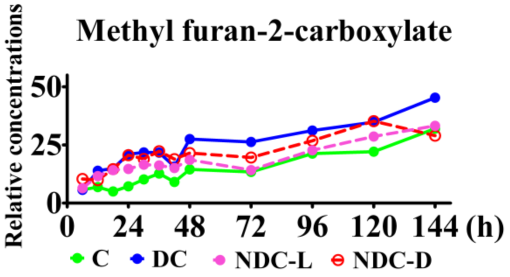
17	(1α,2α,3α)-4-Cyclopentene-1,2,3-triol	$C_5H_8O_3$	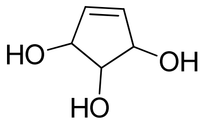	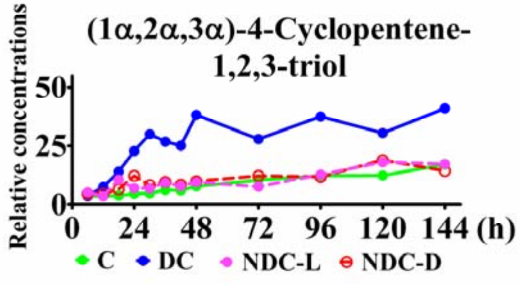
18	Dihydro-4-hydroxy-2(3*H*)-furanone	$C_4H_6O_3$	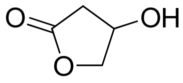	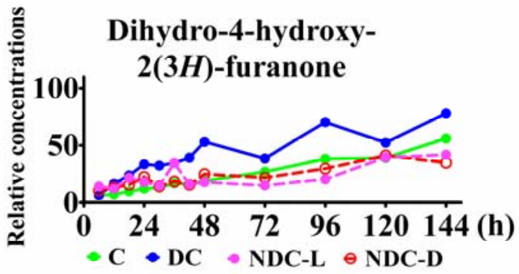

19	2,3-Dihydro-3,5-dihydroxy-6-methyl-4*H*-pyran-4-one	$C_6H_8O_4$	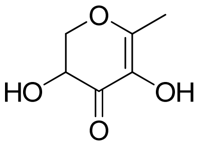	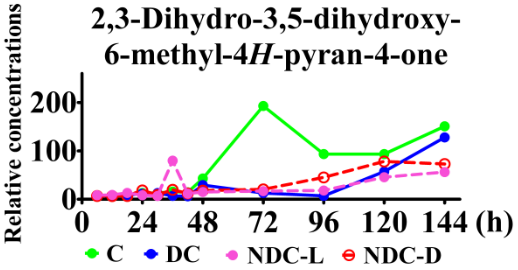
20	1-(3-Ethyltetrahydro-2H-pyran-2-yl	$C_6H_{10}O_2$	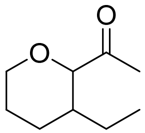	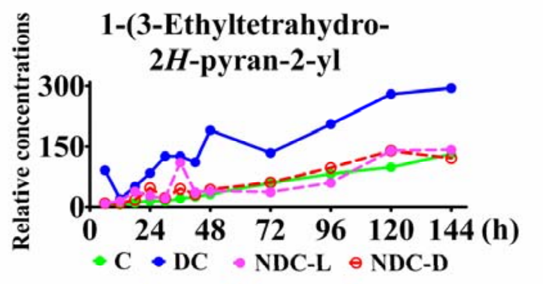
21	5-(Tetrahydro-2*H*-pyran-2-yl)-1-pentanol	$C_{10}H_{20}O_3$	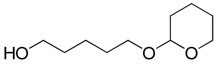	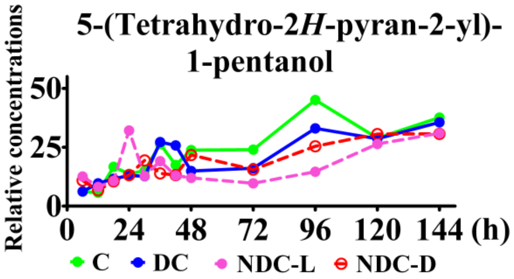

22	Hexahydro-2,6-epoxyfuro[3,2-b]furan-3-ol	$C_6H_8O_4$	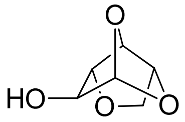	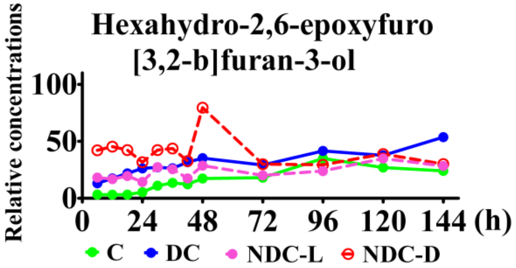
23	(5,5-dimethylheptane-1,3-diyl)dicyclohexane	$C_{21}H_{40}$	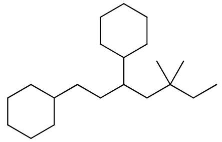	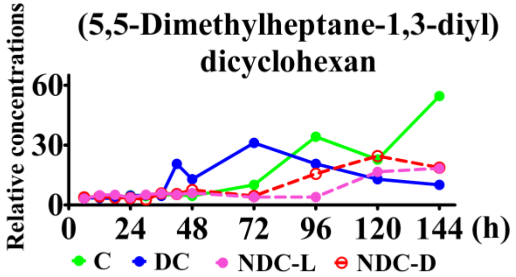
24	Decyl 2-phenylacetate	$C_{18}H_{28}O_2$	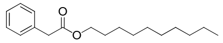	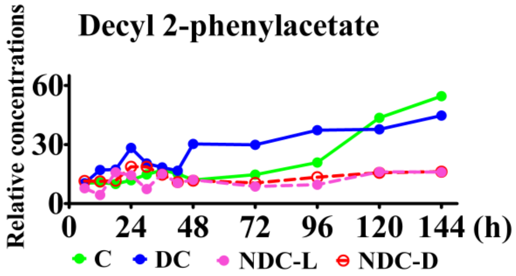

25	Diisobutyl phthalate	$C_{16}H_{22}O_4$	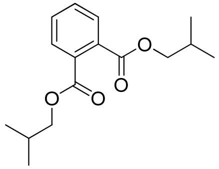	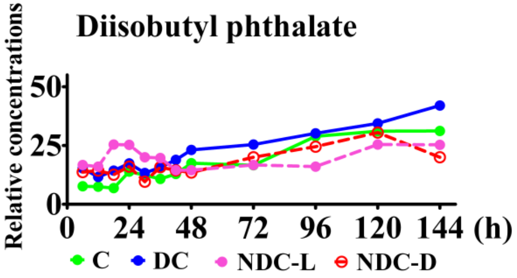
26	2-Ethylhexyl butyrate	$C_{12}H_{24}O_2$	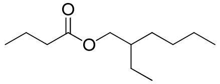	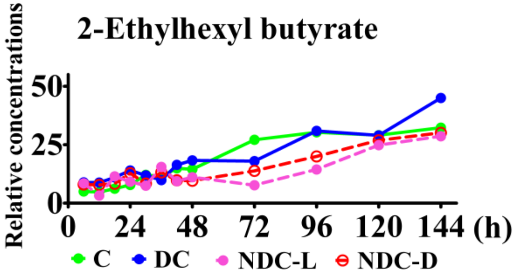
27	Methyl-(3R)-(-)-5-oxo-3-propylpentanoate	$C_9H_{16}O_3$	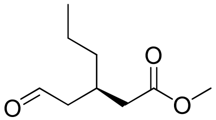	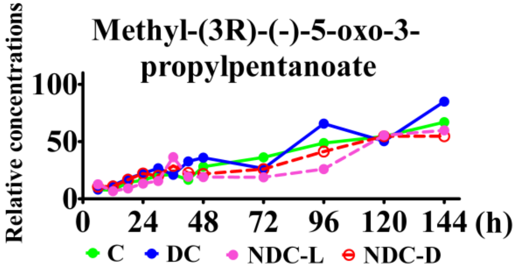

28	Hexadecanoic acid	$C_{16}H_{32}O_2$		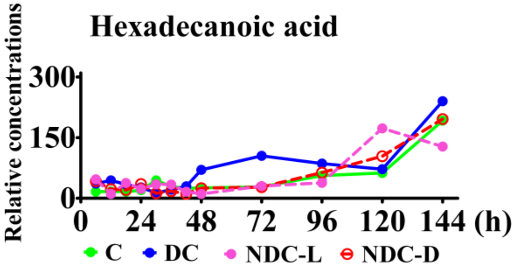
29	Methyl oleate	$C_{19}H_{36}O_2$		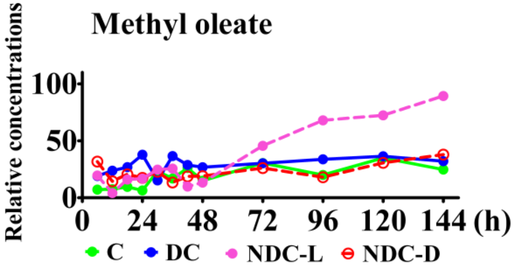
30	(9Z,12Z)-Methyl octadeca-9,12-dienoate	$C_{19}H_{34}O_2$	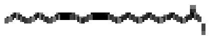	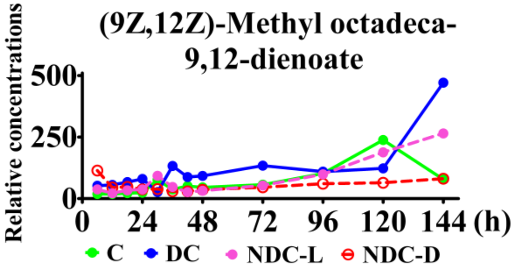

31	(9Z,12Z)-Octadeca-9,12-dienoic acid	$C_{18}H_{32}O_2$	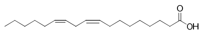	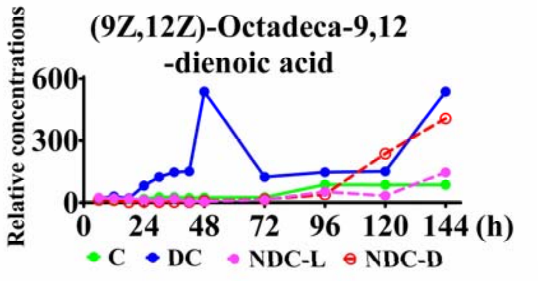
32	3,6,9,12-Tetraoxadolosan-1-ol	$C_{18}H_{38}O_5$	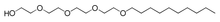	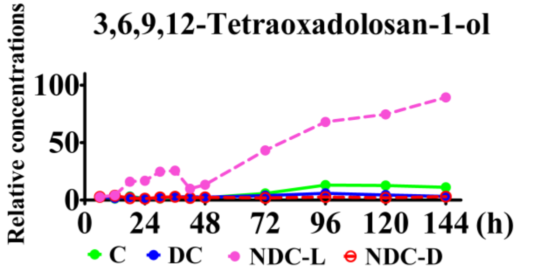
33	6-Methoxy-9*H*-purin-2-amine	$C_6N_7N_5O$	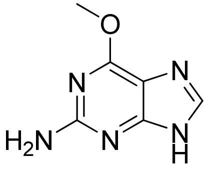	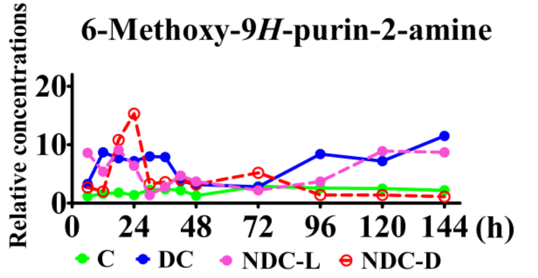

Table S7. GC-MS profiles of metabolites of nematodes under non-direct contact with *A. oligpspora* YMF1.01883 in CMA during 6-144h

No	Rt (min)	Hit Name	Molecular Formula	Structure	Rt (min)
1	9.842	Ethanimidothioic acid	$C_7H_{13}N_3S$	219	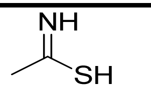
2	9.864	3,6,12-trimethyl-1,4,7,10,13,16-hexaazacyclooctadecane-2,5,8,11,14,17-hexaone	$C_{15}H_{24}N_6O_5$	384	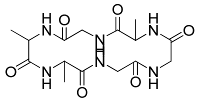
3	10.403	Pentanenitrile	C_5H_9N	83	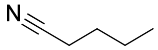
4	12.313	Allantoic acid	$C_4H_8N_4O_4$	176	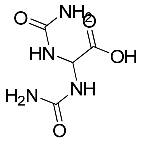
5	25.103	1-（7-hydroxy-8-methoxy-2,3,3a,4,5,5a-11,12-octahydro-H-indolizion	$C_{20}H_{26}N_2O_3$	342	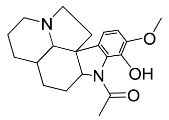
6	6.115	3,5-dimethyl-1,6-heptadien-4-ol	$C_9H_{16}O$	140	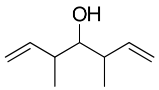
7	6.282	1-butanol	$C_4H_{10}O$	74	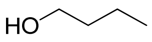
8	8.286	1.2.3.4-butanetetrol	$C_8H_{18}O_4$	178	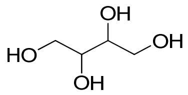

9	9.792	1,2-cycloperlanediol, trans	$C_5H_{10}O_2$	102	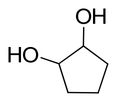
10	9.802	Cis-2ethyl-2-hexen-1-ol	$C_8H_{16}O$	128	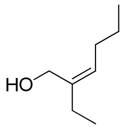
11	10.987	1,2-butanediol	$C_4H_{10}O_2$	90	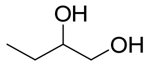
12	15.089	1-octanol,2-butyi	$C_{12}H_{26}O$	186	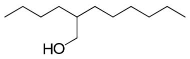
13	18.400	5-Ethyl-3-nonanol	$C_{11}H_{24}O$	172	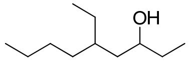
14	19.086	Nonadecanol	$C_{19}H_{40}O$	284	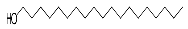
15	20.464	Phenol,2,6-bis-(1,1-dimethylethyl)-4-methyl-	$C_{15}H_{24}O$	220	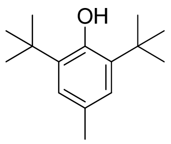
16	21.602	1-hexadecanol, 2-methyl	$C_{17}H_{36}O$	256	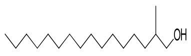
17	30.014	Cholestan-3-ol, 2-methylene	$C_{28}H_{48}O$	400	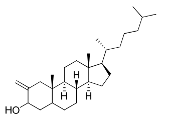
18	3.860	2-pentanone	$C_5H_{10}O$	86	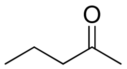
19	3.996	3-Pentanone, 2-methyl-	$C_6H_{12}O$	100	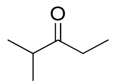
20	14.974	Octadecanal	$C_{18}H_{36}O$	268	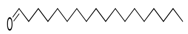

21	22.037	4-hydroxmethylene-2,6-dimethyl-oct-7-en-3-one	$C_{11}H_{18}O_2$	182	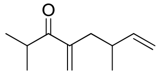
22	2.210	Ethyl methylcarbamte	$C_4H_9NO_2$	103	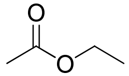
23	8.561	Oxalic acid	$C_{14}H_{24}O_4$	256	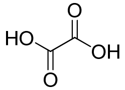
24	9.247	2,3,6,6-tetramethyl-4,5-didehydro-2,3,6,7-tehrahydrothiepine-1-oxiide	$C_{11}H_{18}O_3$	198	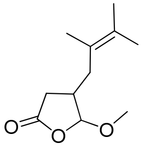
25	9.437	9-octadecendic acid	$C_{18}H_{34}O_2$	282	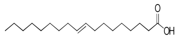
26	9.457	3-Cyclopropylcarbonyioxydodecan	$C_{16}H_{20}O_2$	254	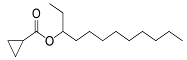
27	9.617	Propanoic acid, 2-hydroxy	$C_4H_8O_3$	104	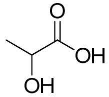
28	10.348	Acetic acid	$C_2H_4O_2$	60	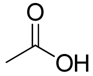
29	12.573	2-furancarboxylic acid	$C_{12}H_{16}O_3$	208	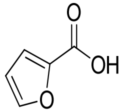
30	18.175	a,в-Crotonolactone	$C_4H_3O_2$	83	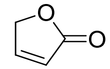
31	19.231	Oxalic acid allyl octadecyl ester	$C_{23}H_{42}O_4$	382	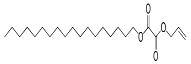

32	19.411	Phthalic acid,<br>butyl undecyl ester	$C_{25}H_{40}O_4$	404	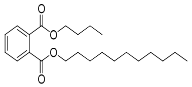
33	19.526	Didodecyl phthalate	$C_{32}H_{54}O_4$	502	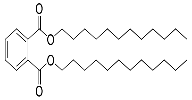
34	19.556	1,2-Bendzenedicarboxylicacid,<br>bis(2-methylpropyl	$C_{16}H_{22}O_4$	278	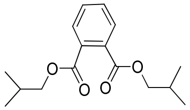
35	19.906	Oxalic acid allyl pentadecyl ester	$C_{20}H_{36}O_4$	340	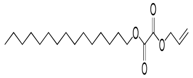
36	20.106	Pentanoic acid,<br>2,2,4-trimethyl-<br>3-carboxyisopropyl isobutyl ester	$C_{16}H_{30}O_4$	286	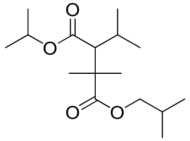
37	21.342	9-octadecenoic acid	$C_{19}H_{36}O_2$	296	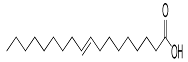
38	21.482	Cyclopentaneundecanoic acid	$C_{16}H_{32}O_2$	254	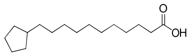
39	21.817	2-octen-1-ol,3,7-dimethyl,isobutyl ester	$C_{14}H_{26}O_2$	226	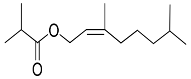
40	22.032	4-hydroxy-4-methyl-hex-5-enoic acid tert-butyl ester	$C_{11}H_{20}O_3$	200	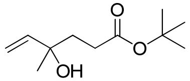
41	22.042	7-methyl-Z-tetradecen-1-ol acetate	$C_{17}H_{32}O_2$	268	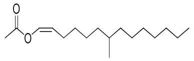
42	22.272	Elcosanoic acid	$C_{20}H_{40}O_2$	312	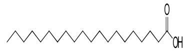
43	22.282	Cyclopentaneundecanoic acid methyl ester	$C_{17}H_{32}O_2$	268	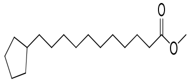

44	22.472	9-Octadecenoic acid	$C_{18}H_{34}O_2$	282	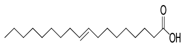
45	24.838	(E)-3-(dodec-2-enyl)dihydrofuran-2,5-dion	$C_{16}H_{26}O_3$	266	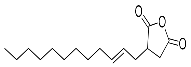
46	22.898	Tetradecanoic acid,<br>12-methylo-	$C_{16}H_{32}O_2$	256	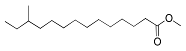
47	24.608	13,16-octadecadiynoic acid	$C_{18}H_{28}O_2$	376	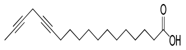
48	24.718	Pentadecanoic acid	$C_{17}H_{34}O_2$	270	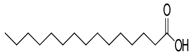
49	25.033	17-octadecynoic acid	$C_{18}O_{32}O_2$	280	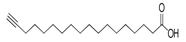
50	27.379	Methyl 9,10-methyl<br>ene-hexadecanoate	$C_{18}H_{34}O_2$	282	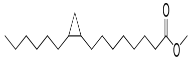
51	28.415	Methyl stearate	$C_{19}H_{38}O_2$	298	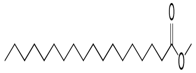
52	28.855	9-octadecenoic acid	$C_{19}H_{36}O_2$	296	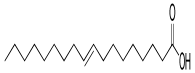
53	29.530	9,12-octadecadienoic acid	$C_{19}H_{34}O_2$	294	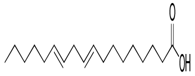
54	29.695	5,8,11,14-eicosatetraenoic acid	$C_{20}H_{32}O_2$	304	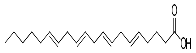
55	7.516	3-Allyloxy-1.2 propanediol	$C_6H_{12}O_3$	132	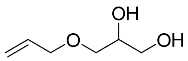
56	9.152	1,3-benzodioxde,<br>2-ethenylhexahydro	$C_9H_{14}O_2$	154	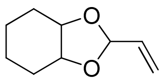
57	15.069	Oxirane octyl	$C_{10}H_{20}O$	156	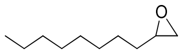

58	16.755	Oxirane, (3,3-dimethylbutyl)-	$C_8H_{16}O$	128	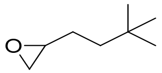
59	16.802	Octadecane, 1-(ethenyloxy)-	$C_{20}H_{40}O$	296	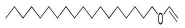
60	20.941	Oxirane, [(dodecyloxy)methyl]	$C_{15}H_{30}O_2$	242	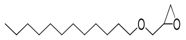
61	22.977	2-tridec-11-ynyloxytetrahydro-2H-pyran	$C_{20}H_{36}O_2$	308	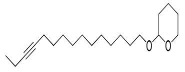
62	16.385	2-furanmethanol	$C_5H_6O_2$	98	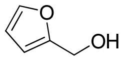
63	6.327	2.23.4-tetramethyl	C_9H_{20}	128	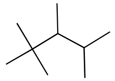
64	6.671	Dodecane	$C_{12}H_{26}$	170	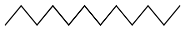
65	7.236	2.4-dimethyl 1.4-pentadiene	C_7H_{12}	96	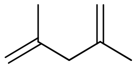
66	12.983	Octane,2,7-dimethyl	$C_{10}H_{22}$	144	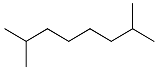
67	18.726	Eicosane	$C_{20}H_{42}$	282	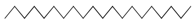
68	19.706	Docosane,11-decyl-	$C_{32}H_{66}$	450	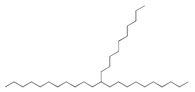
69	19.761	Tetradecane, 2,6,10-trimethyl-	$C_{17}H_{36}$	240	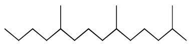
70	21.722	Cyclohexane,1,1,- [1-(2,2-dimethylbutyl)-1,3-propaned	$C_{21}H_{40}$	292	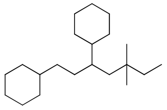

71	22.102	2,6-10-trimethyldodecane	$C_{15}H_{32}$	212	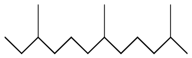
72	19.786	1-Chloyooctadecane	$C_{18}H_{37}Cl$	288	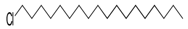
73	22.097	1-Ido-2-methylundecane	$C_{12}H_{25}I$	296	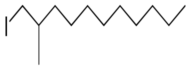

Table S8. HPLC and GC-MS profiles of the five mutants of PKS genes and *A. oligospora* wildtype.

NO.	Gene number	Length (bp)	Function predicted	HPLC	GC-MS
1	AOL_s00043g287 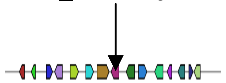	1755	type III polyketide synthases	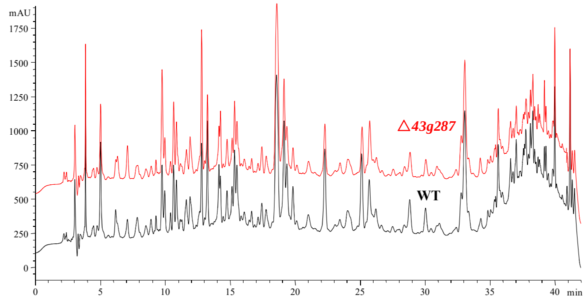	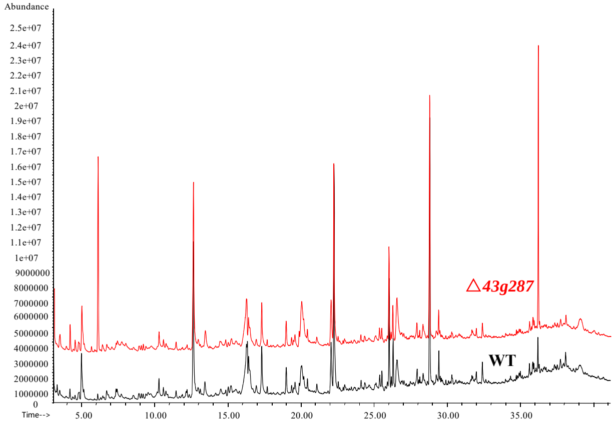
2	AOL_s00043g828 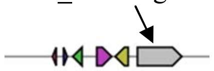	11468	type I polyketide synthases	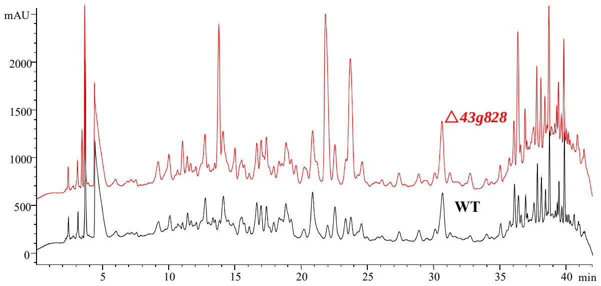	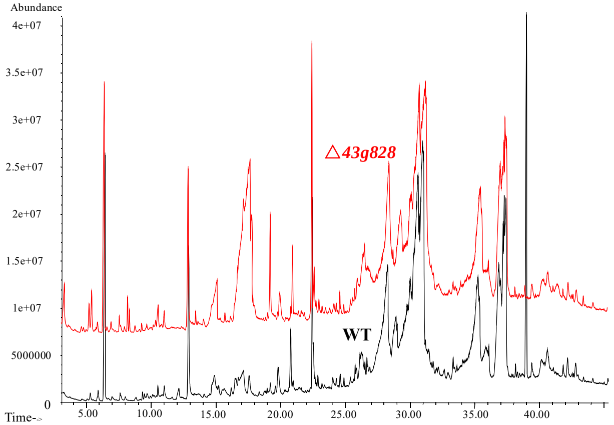

3	AOL_s00079g496 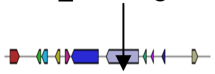	7445	type I polyketide synthases	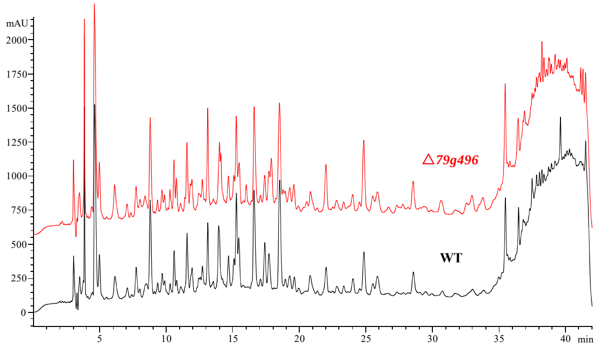	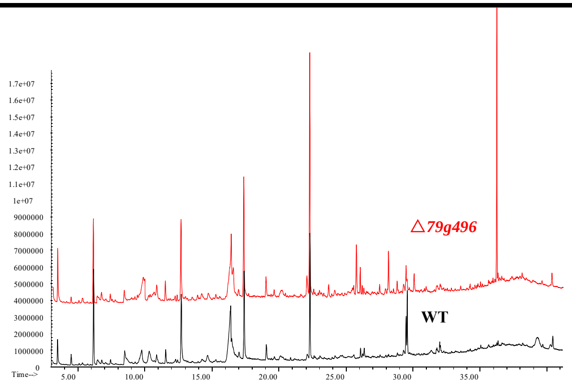
4	AOL_s00215g283 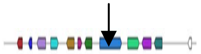	5365	type I polyketide synthases	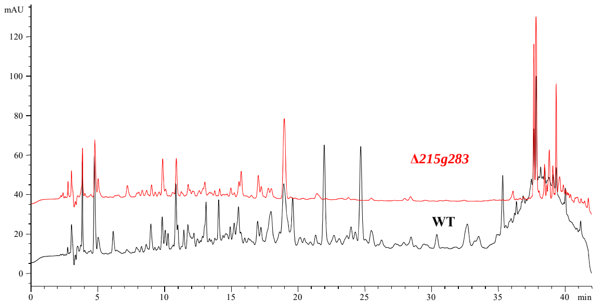	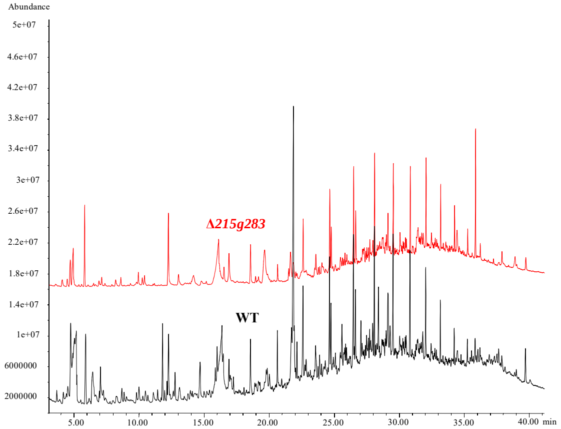

5	AOL_s00215g926	7710	type I polyketide synthases	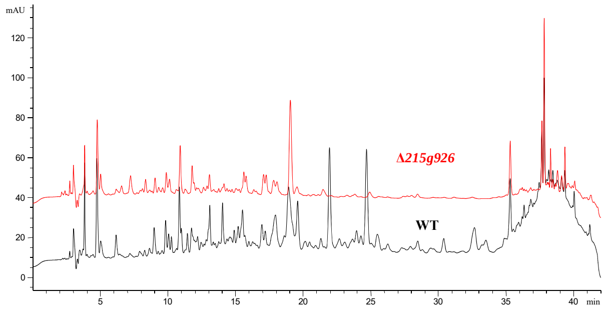	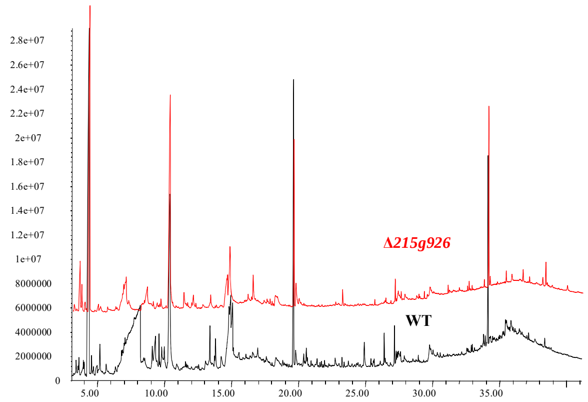

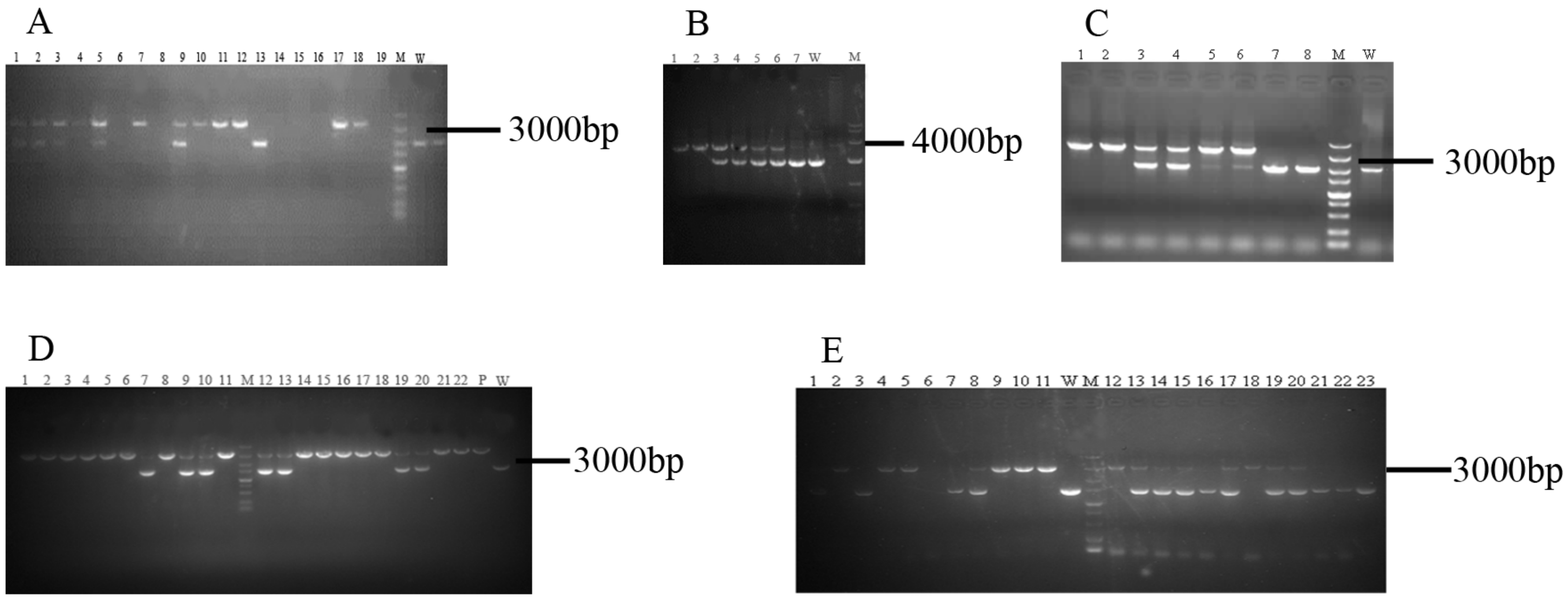

Fig. S2. Five transformants were screened and confirmed by the PCR method. W, wild-type strain; M, marker. (A) lanes 7,10,11,12,17 and 18, six *ΔAOL_s00043g287* mutants. (B) lanes 1 and 2, two Δ*AOL_s00043g828* mutants. (C) lanes 1 and 2, two Δ*AOL_s00215g283* mutants. (D) lanes 1-6, 8, 11, 14-18, 21 and 22, fifteen Δ*AOL_s00079g496* mutants. (E) lanes 2,4,5,9-11,12 and 18, eight Δ*AOL_s00215g926* mutants.

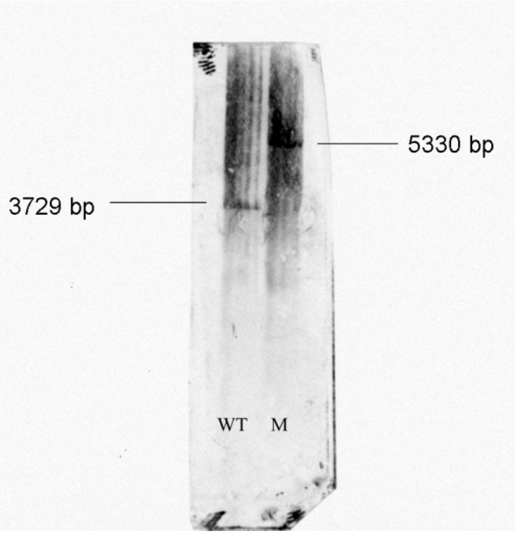

Fig. S3. Southern analysis of the wild-type strain (WT) and the Δ*AOL_s00079g496* mutant (M). The genomic DNA was digested using AgeI.

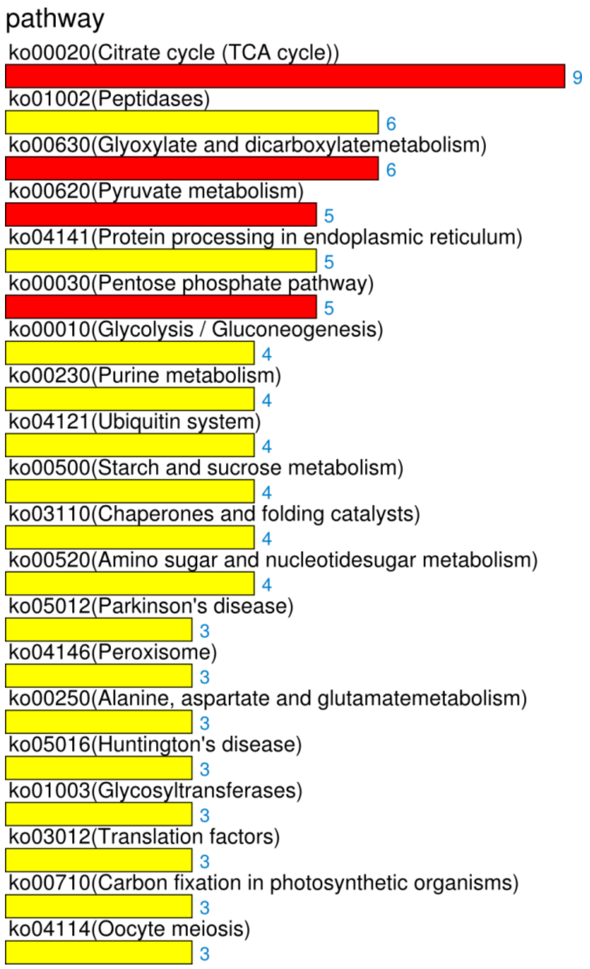
pathway
ko00020(Citrate cycle (TCA cycle))
9
ko01002(Peptidases)
6
ko00630(Glyoxylate and dicarboxylatemetabolism)
6
ko00620(Pyruvate metabolism)
5
ko04141(Protein processing in endoplasmic reticulum)
5
ko00030(Pentose phosphate pathway)
5
ko00010(Glycolysis / Gluconeogenesis)
4
ko00230(Purine metabolism)
4
ko04121(Ubiquitin system)
4
ko00500(Starch and sucrose metabolism)
4
ko03110(Chaperones and folding catalysts)
4
ko00520(Amino sugar and nucleotidesugar metabolism)
4
ko05012(Parkinson's disease)
3
ko04146(Peroxisome)
3
ko00250(Alanine, aspartate and glutamatemetabolism)
3
ko05016(Huntington's disease)
3
ko01003(Glycosyltransferases)
3
ko03012(Translation factors)
3
ko00710(Carbon fixation in photosynthetic organisms)
3
ko04114(Oocyte meiosis)
3

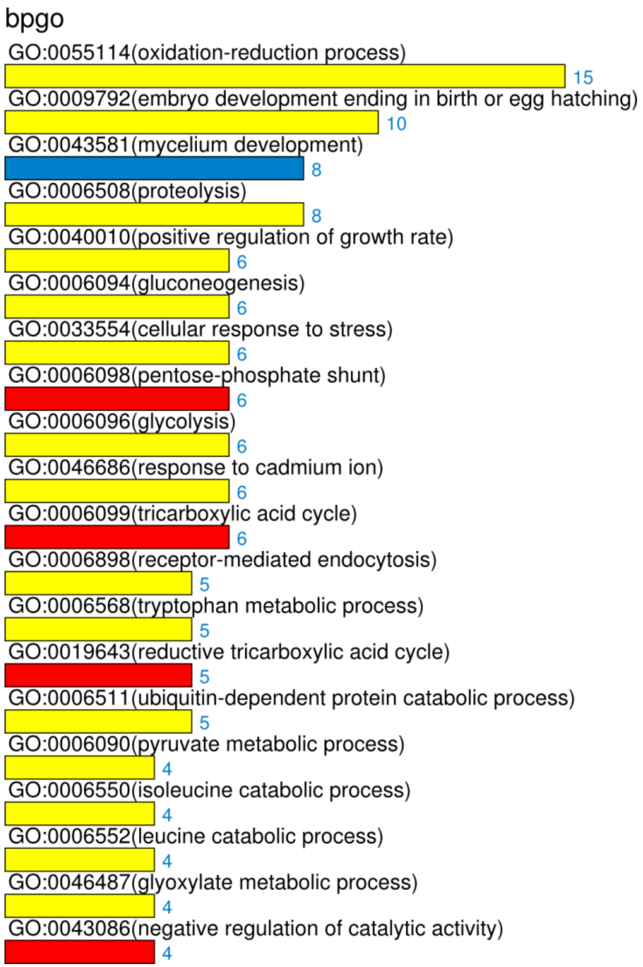
bpgo
GO:0055114(oxidation-reduction process)
15
GO:0009792(embryo development ending in birth or egg hatching)
10
GO:0043581(mycelium development)
8
GO:0006508(proteolysis)
8
GO:0040010(positive regulation of growth rate)
6
GO:0006094(gluconeogenesis)
6
GO:0033554(cellular response to stress)
6
GO:0006098(pentose-phosphate shunt)
6
GO:0006096(glycolysis)
6
GO:0046686(response to cadmium ion)
6
GO:0006099(tricarboxylic acid cycle)
6
GO:0006898(receptor-mediated endocytosis)
5
GO:0006568(tryptophan metabolic process)
5
GO:0019643(reductive tricarboxylic acid cycle)
5
GO:0006511(ubiquitin-dependent protein catabolic process)
5
GO:0006090(pyruvate metabolic process)
4
GO:0006550(isoleucine catabolic process)
4
GO:0006552(leucine catabolic process)
4
GO:0046487(glyoxylate metabolic process)
4
GO:0043086(negative regulation of catalytic activity)
4
